# *Mycodnaviridae* is a clade of giant viruses that persistently infect zoosporic fungi

**DOI:** 10.1371/journal.pbio.3003937

**Published:** 2026-08-25

**Authors:** Jillian M. Myers, Frederik Schulz, Saleh Rahimlou, Vikas Yadav, Kevin R. Amses, David Rabern Simmons, Sheng Sun, Michelle Orozco-Quime, Joseph Heitman, Jason E. Stajich, Timothy Y. James

**Affiliations:** 1 Ecology and Evolutionary Biology, University of Michigan, Ann Arbor, Michigan, United States of America; 2 DOE Joint Genome Institute, Lawrence Berkeley National Laboratory, Berkeley, California, United States of America; 3 Molecular Genetics and Microbiology, Duke University School of Medicine, Durham, North Carolina, United States of America; 4 Microbiology and Plant Pathology, University of California, Riverside, Riverside, California, United States of America; 5 University of Michigan Herbarium, Ann Arbor, Michigan, United States of America; Stowers Institute for Medical Research, UNITED STATES OF AMERICA

## Abstract

Giant viruses of the phylum *Nucleocytoviricota* have emerged as particularly notable due to their increasingly recognized impacts on eukaryotic genome evolution. Their origins are hypothesized to predate or coincide with the diversification of eukaryotes, and they have been detected in hosts that span the eukaryotic tree of life. But surprisingly, such viruses have not been definitively found in Kingdom Fungi, though earlier genomic and metagenomic work suggests putative associations. Here we report both “viral fossils” and active infection by giant viruses in fungi, particularly in the zoosporic phyla Blastocladiomycota and Chytridiomycota. The recovered viral assemblies span up to 350 kb, encode over 300 genes, and form a monophyletic family-level clade within the *Nucleocytoviricota* related to orders *Imitervirales* and *Algavirales*, which we name *Mycodnaviridae*. We observed variation in infection status among the isolates including apparent active infection and transcriptionally suppressed states, suggesting that viral activation may be constrained to certain life stages of the host. Our experimental findings add to the limited natural virus-host systems available in culture for the study of giant viruses and expand the known host range of *Nucleocytoviricota* into a new kingdom that contains many model species. *Mycodnaviridae* have a global distribution, which invites inquiry into the implications of these infections for host traits, host genome evolution, and the metabolic impacts on ecosystems.

## Abstract 2

Giant viruses are known across eukaryotes, yet definitive evidence of fungal infection has been missing. This study identifies Mycodnaviridae, a globally distributed family of giant viruses that persistently infect zoosporic fungi, providing a promising model for studying giant virus infection in eukaryotes.

## Introduction

In the entire Fungal Kingdom, only a handful of DNA viruses are known—all small circular single-stranded (CRESS) viruses [1, 2]. This apparent rarity of DNA viruses in fungi likely results from taxonomic biases in searches, rather than a true biological phenomenon. The study of mycoviruses has historically focused on their application for biological control of fungal pathogens, particularly in agricultural contexts, and as a result most research has involved viruses infecting fungi in the Ascomycota—one of two phyla comprising the subkingdom Dikarya, which contains over half of described fungal taxa. The current fungal taxonomic division includes eight phyla of zoosporic fungi that reproduce with motile spores and represent a particularly understudied paraphyletic assemblage that have retained ancestral characters shared with animals [3,4]. We previously demonstrated that by expanding the taxonomic scope of considered fungal hosts to include these lineages outside of the Dikarya the known RNA mycoviral diversity was greatly increased [5]. However, the presence of DNA viruses in these fungi has been almost entirely unexplored (but see [6]. Indeed, the finding of giant viruses (GVs), which we report here, began as a serendipitous discovery resulting from a large genomic sequencing effort across these fungal phyla [7].

Nucleocytoviricota is a phylum of highly diverse “giant viruses” including those with the largest and most complex genomes yet known, which is a result of gene acquisition from eukaryotic hosts and bacteria as well as substantial gene duplication in some lineages [8]. Since their discovery GVs have challenged the established expectations in virology as some encode genes involved in transcription and translation [9–11] as well as various metabolic processes [12–14]. Studies have shown that up to 10^5^ GV genomes can exist within a single microliter of ocean water [15] and the taxon richness of just one order, *Imitervirales*, surpasses that of both bacteria and archaea in oceans [16].

Despite their incredible abundance, relatively few GVs have been linked to a host, and only a scant number of natural GV-host systems have been studied in the laboratory. Much of what is known about giant virus biology has been learned from protist-GV interactions. Lytic dynamics are common wherein following endocytosis of the virus into the host cell the viral DNA is transported to the nucleus for replication, then to cytoplasmic viral factories for assembly, and virions exit the cell by budding or cell lysis. Interestingly, some GVs in the Algavirales can also enter a latent phase wherein the viral genome is integrated into a host chromosome. Reactivation of the proviruses can be triggered by temperature, but the molecular mechanisms involved are unknown (reviewed in [17]. GVs must certainly have meaningful ecosystem-level biogeochemical consequences which have yet to be fully understood or quantified. The development of additional laboratory model systems of GVs and their natural hosts could lead to advances in our understanding of virus–host interactions, including host immune response, viral “reprogramming” of host cells, metabolic impacts, and their ecosystem-level consequences.

Confirmed associations with GVs include hosts from a breadth of taxa—from single-celled algae to invertebrates to animals (reviewed in [18])—and metagenomic- and metatranscriptomic-powered inferences suggest an even broader scope of eukaryotic hosts [19,20]. Previous studies have suggested associations between *Nucleocytoviricota* and fungi through the identification of putative fungal homologs in metagenomic-assembled giant viruses [19,21,22] and giant virus endogenous viral elements (EVEs) in fungal genomes [23–26]. Of these mentioned, only the EVE studies directly connect fungi with viruses, while the metagenomics studies predict fungal affiliation of the viruses based on gene homology and, in the case of [22], the dominance of fungi in the environment. The latter does not preclude the possibility of non-fungal hosts. Active GV infections in fungi have not previously been documented nor have novel clades that include active giant viruses in fungi been characterized; we describe both here and present a promising new model system to further examine GV–fungal interactions. The cumulative evidence suggests a rich history between GVs and fungi, with much more yet to be understood.

## Results

### Zoosporic fungi contain giant virus genes and genomes with global distribution

We identified homologs of the giant virus major capsid protein (MCP; NCVOG0022) in the genomes of 17/141 fungi with HMM searches (S1 Fig and S1 Table). The distribution of MCP homologs is restricted to non-Dikaryotic lineages, and, particularly, the zoosporic fungi; no MCPs were found by hmmsearch in non-zoosporic lineages (*n* = 40). We further searched all fungal genomes in Mycocosm [27]; *n* = 2,543) for MCP homologs by blastp and protein model searches, which resulted in one hit to a non-zoosporic fungal species: *Dimargaris cristalligena* (phylum Zoopagomycota), a mycoparasite. Among the zoosporic lineages, MCPs were found in Chytridiomycota (*n* = 12), Monoblephidomycota (*n* = 2), and Blastocladiomycota (*n* = 3). Blastocladiomycota are a particularly poorly sampled group, and thus our genomic searches were limited (*n* = 7). However, MCPs appear to be common in the most highly sampled genus within this phylum, *Allomyces.* Based on available sequences, we designed primers targeting the MCP gene of *Allomyces* and found 30 out of 58 unique isolates (51.7%) were positive for capsid by PCR.

**Fig 1:**
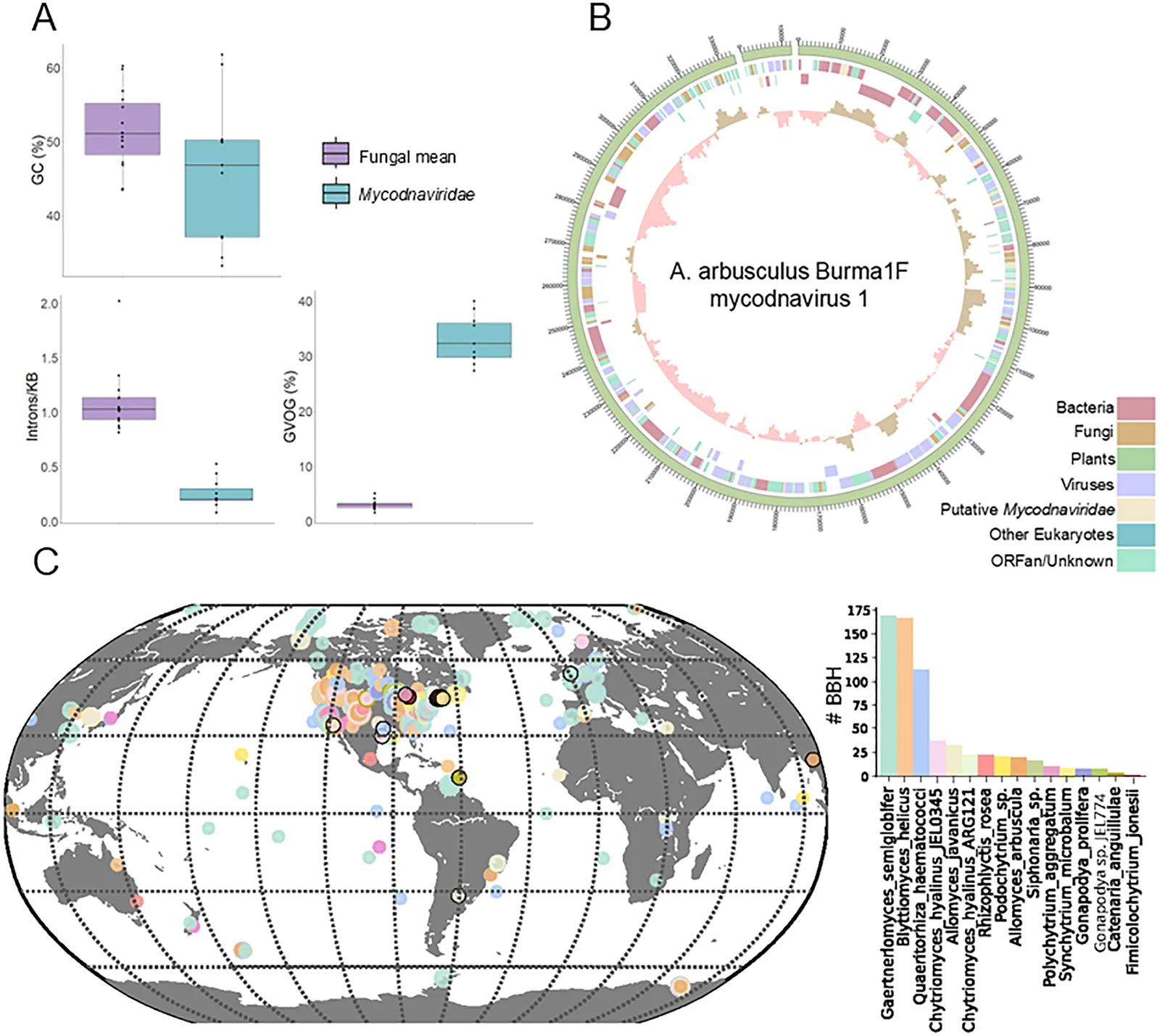
Genomic features of *Mycodnaviridae.* **(A)** Boxplots of genome statistics for the fungal genomes found to contain MCP genes (*n* = 17): mean values across all contigs (“Fungal mean”, purple), and MCP contigs that met the criteria set for orthology analysis (ViralRecall score >2, viral region length >70%, intron density >25% different from host; “*Mycodnaviridae”*, teal). **(B)** The genome of Allomyces arbusculus Burma1F mycodnavirus 1 is a mosaic of bacteria; viral, and eukaryotic genes. Contigs are represented by the outermost ring in the circularized genome plot. From outside to inside: ORFs on the forward strand, ORFs on the reverse strand (both color-coded by provenance), GC skew. **(C)** Global distribution of the *Mycodnaviridae-*like sequences based on the major capsid protein. Colored circles correspond to metagenomic MCP sequences with best blast hit (BBH) to *Mycodnaviridae* identified in this study and circles with a black outline indicate geographic location of *Mycodnaviridae* and hosts described in this study. This map was made using the basemap from matplotlib mpl_toolkits with the ‘robin” projection. The data underlying this Figure can be found in https://doi.org/10.7302/p5ay-ng61.

The contigs containing MCP genes vary widely among the fungal isolates in gene content, GC composition, intergenic distance, and other metrics, suggesting numerous independent endogenization events (Fig 1A). In three isolates (*Podochytrium* sp. JEL797, *Gaertneriomyces semiglobifer* Barr43, and *Fimicolochytrium jonesii* JEL569), MCP was the only gene of viral provenance on contigs which otherwise contain primarily fungal genes and have GC content and intron density similar to other host contigs (S2 Fig). These MCPs may be the result of horizontal gene transfer (HGT) from GVs, or remnants from past latent GV infections. In other isolates, contigs with MCPs appear as mosaics of viral, prokaryotic, and fungal genes, which include multiple additional giant virus orthologous genes (GVOGs), and have markedly greater gene density compared to host contigs, consistent with reference GV genomes (Figs 1A, 1B, and S2). These giant virus-like contigs range up to nearly 350 kb and are suggestive of either very recent endogenization or, as we later evidence, proviral (latent) state. Interestingly, in five isolates the MCP contig contains a gene encoding an integrase with sequence similarity to those of polinton-like viruses (*Eupolintoviridae*). Polintons are dsDNA transposons of eukaryotes with ancient and enigmatic associations with GVs [28]. Our finding of polinton-like integrases coinciding with nucleocytoviricot-like polB and other GVOGs may help elucidate the relationships of these groups.

Based on MCP data previously extracted from global metagenomics datasets, we assessed the global distribution of these novel fungal MCPs. Environmental MCP homologs with bidirectional best blast hits to fungal MCPs have a global distribution (Fig 1C). The *Allomyces* isolates tested by PCR for MCP have worldwide provenance, thus also suggestive of a long history of viral infection.

We first closely analyzed the GV genome assemblies from two strains of *Allomyces*. A. arbusculus Burma1F mycodnavirus 1 includes two contigs of total size 345,111 bp, encoding 314 genes with likely provenance, based on homology, assigned as follows: 10% fungi, 14% bacteria, 20% virus, 4% putatively *Mycodnaviridae-*specific, and 52% unknown/ORFan (Fig 1B). The isolate *A. javanicus* California12 has multiple large contigs (ranging between 80 and 288 kb) with giant virus signatures (S3 Fig). Interestingly, when binned by tetramer frequency, the viral contigs in *A. javanicus* California12 form two bins suggesting the presence of two distinct viruses. The two bins are roughly similar in size (212,591 bp and 287,844 bp) and show some duplication of hallmark GVOGs: each bin contains copies of MCP, D5-like helicase-primase, DNA PolB, mRNA capping enzyme large subunit, and superfamily II helicase. The larger bin contains an additional five GVOGs (YqaJ recombinase, RNA ligase, disulfide (thiol) oxidoreductase, transcription initiation factor IIB, and A32-like packaging ATPase). *A. javanicus* strains are believed to be hybrids [29] and the two viruses could have originated from the two parents of this diploid sporophyte. Alternatively, this finding could be a result of co-infection by two distinct virus species.

### *Mycodnaviridae* is a monophyletic clade of fungal giant viruses

**Fig 2:**
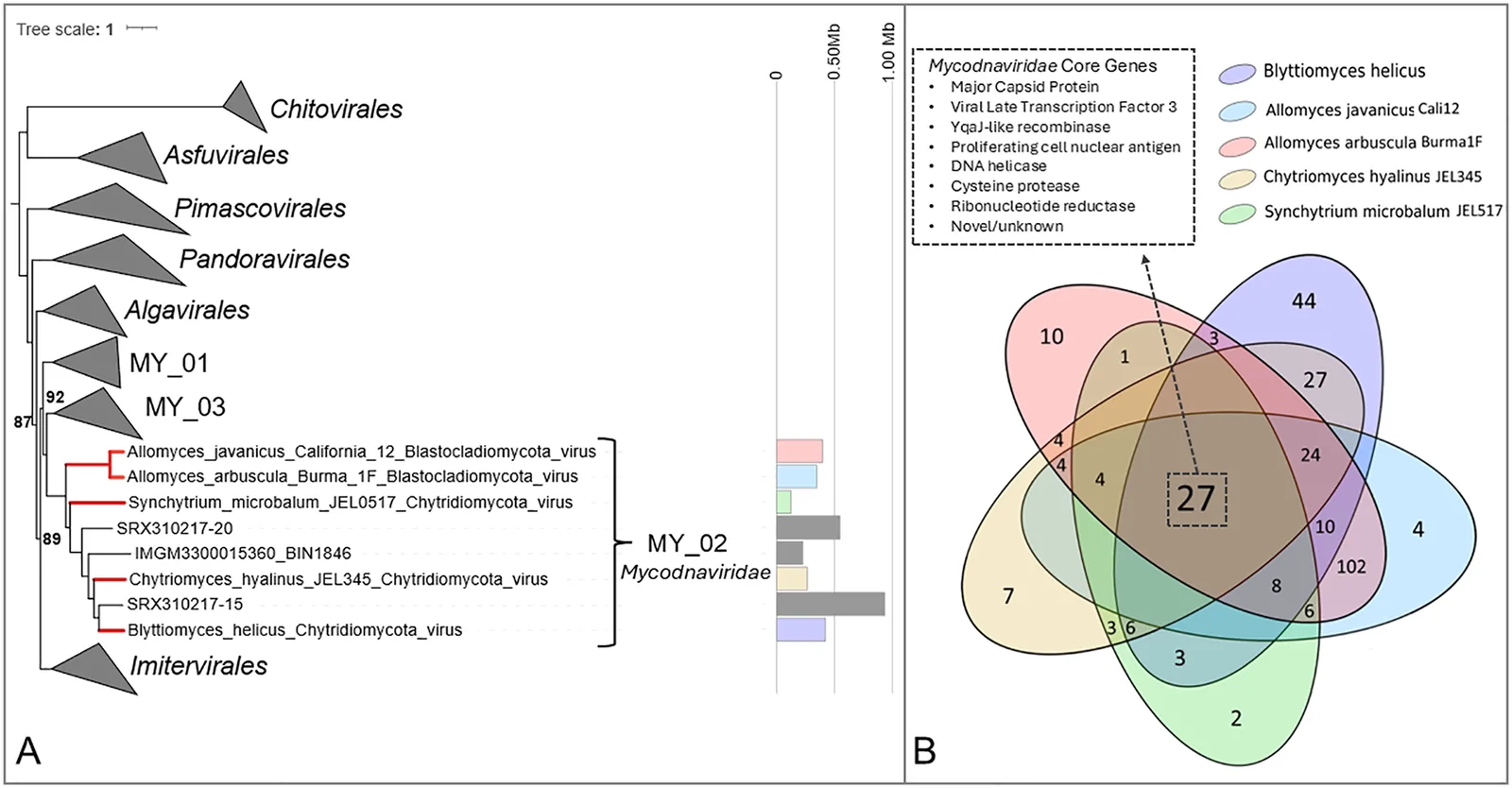
Phylogenetic position of the *Mycodnaviridae* and core genes. **(A)** Position of the *Mycodnaviridae* clade in the species tree of the *Nucleocytoviricota*, wherein the un-collapsed branches represent *Mycodnaviridae*, and MY_01-03 are as in Vasquez and colleagues [31]; within this clade, branches highlighted in red indicate those genomes sequenced from fungi, and branches in black indicate metagenomics-assembled genomes previously known as IM_02. The tree is rooted at Pokkesviricetes. Bars to the right indicate recovered assembly sizes. **(B)** Venn diagram indicating the number of orthologous gene clusters shared between the 5 most complete *Mycodnaviridae* genomes. The data underlying this Figure can be found in https://doi.org/10.7302/p5ay-ng61.

An updated species tree of representative *Nucleocytoviricota*, based on a concatenated alignment of the 7 protein GVOG7 [30] showed that five of the 12 fungal-associated EVEs contained sufficient marker recovery (≥3/7 GVOGs) for inclusion in the final tree. These genomes formed a monophyletic clade with the three metagenome-assembled genomes (GVMAGs) previously assigned to IM_02, indicating that the former IM_02 lineage is expanded and here treated as MY_02 or *Mycodnaviridae* (Fig 2A; [31]. In the revised phylogeny, *Mycodnaviridae* is recovered within a broader order-level clade basal to *Imitervirales*, consistent with the taxonomic framework proposed by Vasquez and colleagues [31]. This supports the main conclusion that fungal giant viruses cluster with the former IM_02 lineage. Hosts of this lineage are unknown, but the clustering of *Mycodnaviridae* in this clade may support a fungal association for the other GVMAGs in MY_02.

We investigated whether a core suite of genes exists that functionally define the *Mycodnaviridae* by conducting orthology analysis on the most complete GV assemblies–five have at least 9 of the 20 core GVOGs present in the majority of the *Nucleocytoviricota* [32]. This resulted in 27 gene clusters shared by all five (Fig 2B) though this is likely an underestimate due to variation in assembly completeness, which is suggested by the variation in GV assembly sizes recovered from different fungal hosts. In addition to *Nucleocytoviricota* core GVOGs such as MCPs, VLTF3, and YqaJ-like recombinase, the *Mycodnaviridae* core set is composed of gene orthogroups involved in DNA biosynthesis such as DNA helicase, ribonucleotide reductase, and proliferating cell nuclear antigen, which are also GVOGs found in other large and giant viruses. Other shared gene clusters include a cysteine protease, protein with N-acetylglucosaminyltransferase activity (possibly involved in cell wall modification), six genes of unknown function, and five novel genes apparently unique to these viruses.

### *Allomyces* viruses are integrated in the nuclear genome and show variation in methylation modification patterns

Critical questions about the giant virus elements are where the apparently complete elements are in the genome and whether and when they are capable of replicative infection. We conducted long-read whole-genome sequencing by Oxford Nanopore to determine whether the viral contigs previously recovered are contiguous in the host genome and to detect methylation modification. We also conducted RNA-Seq to assess expression of viral genes and quantitative PCR of MCP genes as a proxy for viral titer.

**Fig 3:**
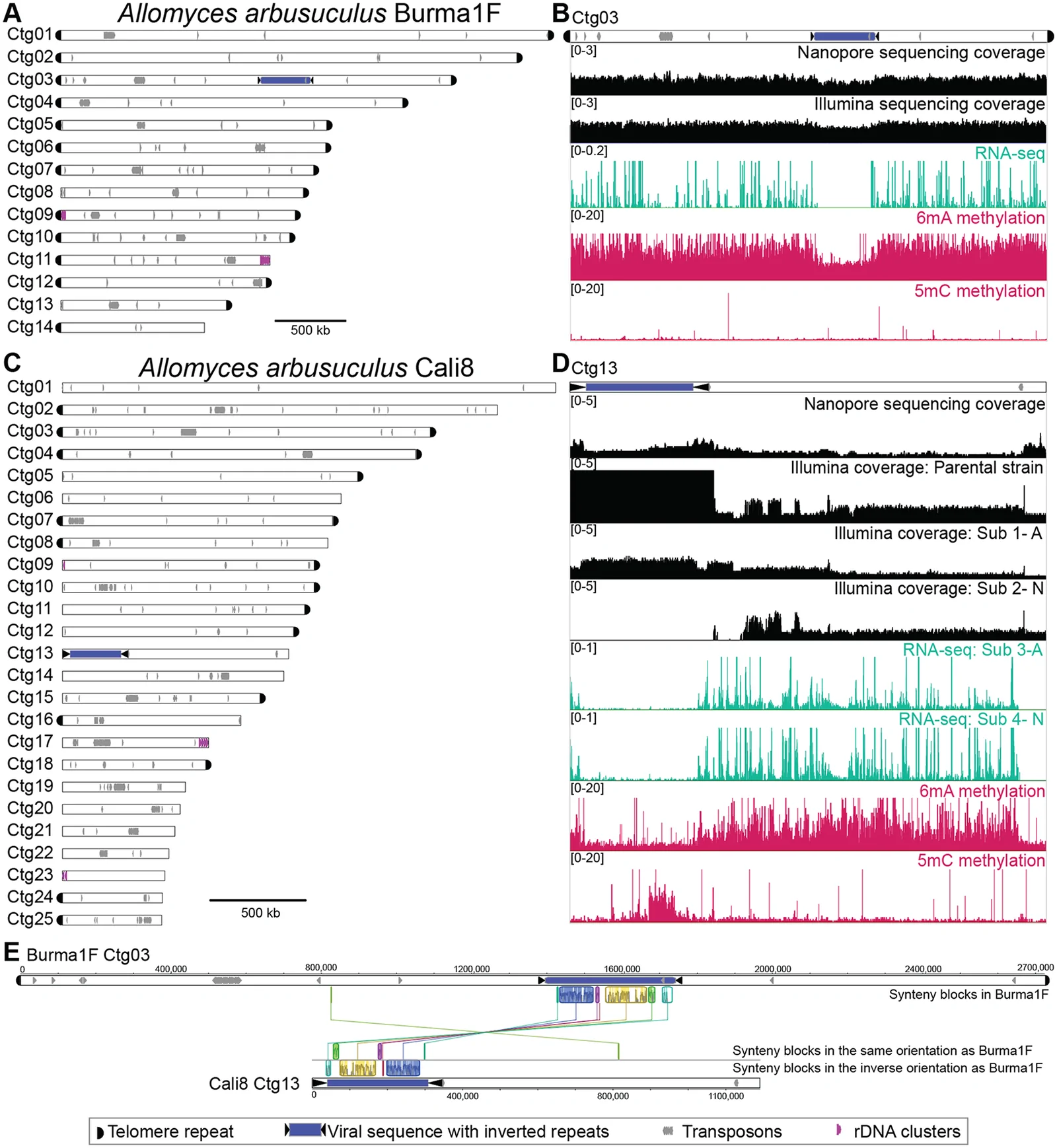
*Allomyces* viruses are integrated into the nuclear genome, with complex patterns of expression/suppression and methylation modification. **(A)** Nanopore-based assembly of *A. arbusculus* Burma1F shows the virus integrated into the host nuclear genome (Ctg 03, blue). **(B)** Mapping of Nanopore reads, Illumina sequencing reads, RNAseq reads, 6mA and CpG methylation extracted from Nanopore sequencing reads across the virus-harboring contig of *A. arbusculus* Burma1F. Note that the copy number of the viral region for JM-6 exceeds the y-axis displayed, see S4 Fig. **(C)** Nanopore-based assembly of *A. arbusculus* Cali8 shows the virus integrated into the host nuclear genome (Ctg 13, blue). **(D)** Mapping of Cali8 Nanopore reads, Illumina sequencing reads of original strain (“Parental strain”) and subculture strains with abnormal (“Sub 1-A”) and normal phenotype (“Sub 2-N”), RNAseq reads of a Cali8 strain with abnormal (“Sub 3-A”) and normal phenotype (“Sub 4-N”), and 6mA and CpG methylation extracted from Nanopore sequencing reads across the virus-harboring contig of *A. arbusculus* Cali8. **(E)** Viral insertion regions of B1F and Cali8 are largely not syntenic, while the viruses themselves are highly syntenic with regions in inverted orientation. In (B) and **(D)**, the bracketed numbers denote the y-axis scale. Nanopore and Illumina sequencing data are shown as copy number (read coverage normalized by average genome coverage), RNA-seq data is shown as RPKM (normalized per-base coverage as bins per million reads), and methylation data as fraction of reads methylated. The data underlying this Figure can be found in NCBI BioProject PRJNA1257431.

In *Allomyces arbusculus* Burma1F, long-read sequencing revealed the viral contigs side-by-side and integrated into a chromosome that harbors telomere repeats on both ends (contig 03: 1397320-1743578; Fig 3A and 3B). Nanopore-based genome assembly also revealed 13 clusters of LTR-transposons, only one per contig, that potentially represent centromeres similar to those observed in the dikaryotic fungus *Cryptococcus neoformans* [33, 34]. With interest in whether the viral genes are expressed and, if so, whether expression occurs throughout host tissue or is constrained to some structures or life cycle stages, we sequenced RNA of *A. arbusculus* Burma1F at three different stages. We found almost no evidence of expression from the viral genes, with the exception of genes in the flanking region (Fig 3B). CpG methylation was not detected in these viral regions per the Nanopore-generated data but, curiously, 6mA methylation was reduced relative to the rest of the chromosome (Fig 3B). Thus, while the expression of A. arbusculus Burma1F mycodnavirus 1 was apparently silenced at the time of sequencing, the mechanism is as yet unknown.

We performed both short- and long-read sequencing of an additional *Allomyces* strain, *A. arbusculus* Cali8, and observed a different pattern. Though the Illumina assembly was more fragmented for Cali8 than Burma1F, viral contigs were detected that harbored MCPs, seven additional core GVOGs, and a viral-like integrase. Interestingly, these contigs averaged nearly 50-fold higher coverage than the rest of the genome (Fig 3D, “Parental Strain”; S4 Fig), suggesting that these DNAs were in high titer at the time of sequencing. When mapped onto the nanopore-based assembly, these high-coverage contigs were side-by-side (Fig 3C), and flanked by additional high-coverage regions on either side for a total GV assembly size of approximately 350 kb. Like Burma1F, 6mA methylation was detected across the viral region at a lower rate than the rest of the genome (Fig 3D); but in contrast to patterns in Burma1F, CpG methylation was detected in parts of the viral region (Fig 3D).

We compared the host genomic regions of viral sequence integration in Cali8 versus B1F. We found only one high-confidence syntenic region >1 kb (Fig 3E). Both endogenous viruses harbor inverted terminal repeats. Within a species, these repeats were >99% identical on either side; however, the sequences are not conserved between species. The repeats on the termini of A. arbusculus Burma1F mycodnavirus 1 are approximately 9 kb, while the repeats on the termini of A. arbusculus Cali8 mycodnavirus 1 are an astonishing 34 kb. The viral regions themselves overall are syntenic, although seem to have undergone significant structural rearrangements with large syntenic blocks in the inverse orientation between the two isolates (Fig 3E).

### Evidence for viral replication and disease phenotype

Our initial clue that viral replication was occurring in Cali8 was based on high coverage of viral contigs in our short-read sequencing data. We reisolated this strain from our stock of resting sporangia and attempted to trigger viral replication, assessed the morphology for evidence of a diseased phenotype, re-sequenced the genomes, performed RNA-Seq, and conducted quantitative-PCR assays to assess viral gene abundance.

**Fig 4:**
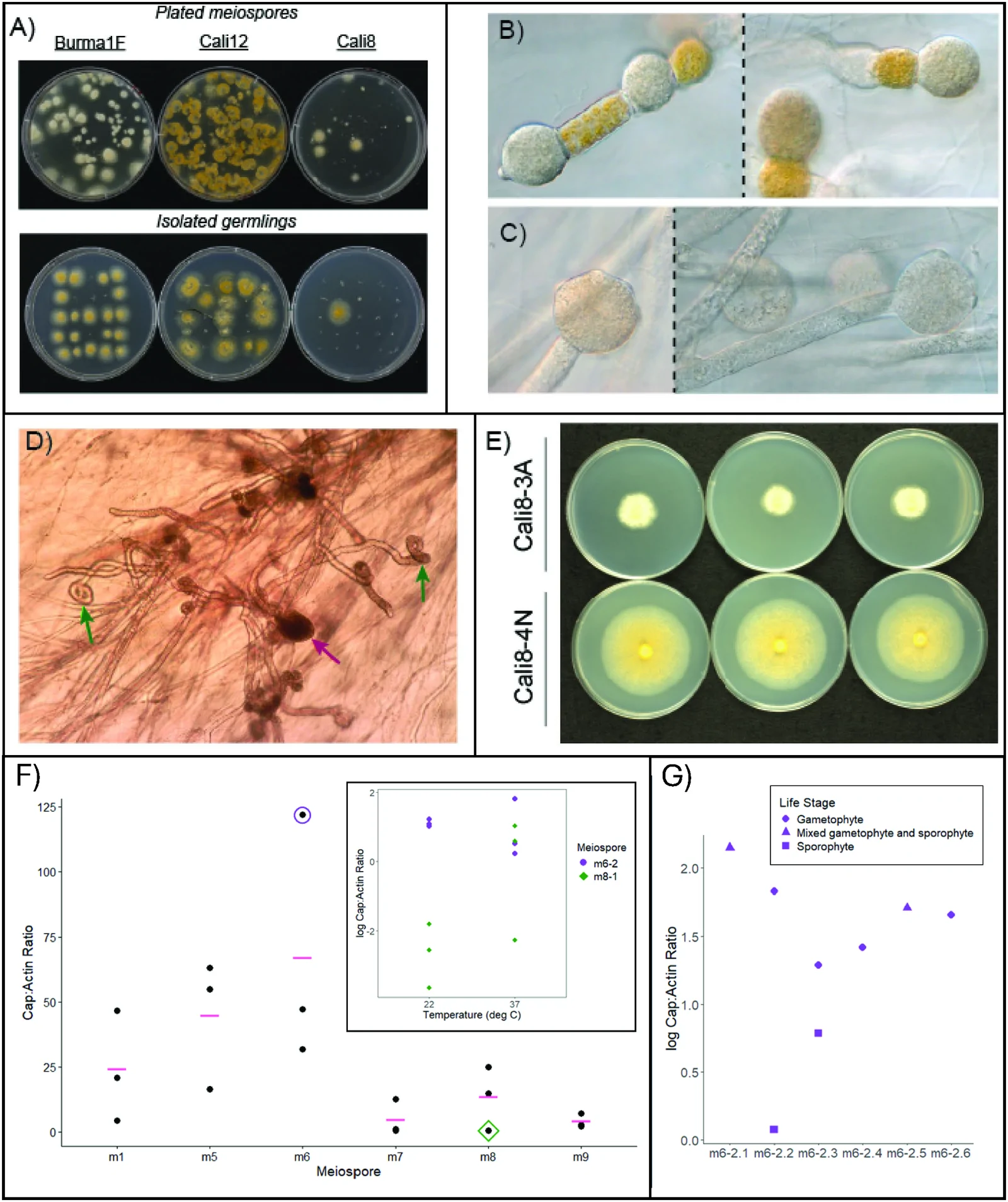
Phenotypic and viral titer differences between isolates of Allomyces across lifestages. **(A)** Meiospores of three *Allomyces* isolates show differences in germination rates **(B)** Cali8-N subcultures display normal gametophyte phenotype. **(C)** Cali8-A subcultures display malformed gametophytes with swollen and unproductive male gametangia. **(D)** Unusual twisted hyphae (green arrows) and blebbing (pink arrow) in Cali8-A. **(E)** Cali8-A has a reduced growth rate relative to Cali8-N. Bottom panel shows qPCR results as the ratio of MCP to actin DNA copies, a proxy for viral abundance. **(F)** Isolates grown from individual meiospores from the same parent varied significantly (*p* < .001) in MCP:actin ratio (individual data points are shown as black dots, means are shown as pink bars). An isolate with very high viral abundance (m6-2, purple circle) and very low viral abundance (m8-1, green diamond) were subcultured at 22 and 37 °C (inset). **(G)** M6-2 was further subcultured and MCP:actin ratio assessed at various life stages. The data underlying this Figure can be found in https://doi.org/10.7302/p5ay-ng61.

Fungi in the phylum Blastocladiomycota, such as *Allomyces*, have life cycles that include the alternation of generations as haploid gametophytes (which produce haploid gametes) and as diploid sporophytes (which produce diploid spores) (S5 Fig). During the sexual phase of the typical healthy culture of *Allomyces* a haploid meiospore germinates to produce a gametophyte with pairs of “male” and “female” reproductive structures (gametangia), each producing gametes which fuse to ultimately form a diploid sporophyte. The mature sporophyte can continue growth in a diploid asexual cycle and also produce resting sporangia that undergo meiosis to produce haploid meiospores which develop into gametophytes after a brief motile period. We found that gametophytes of Cali8, unlike those of Burma1F and Cali12, showed a high rate of mortality and morphological and growth rate variation (Fig 4A). Some gametophytes had typical growth and development with terminal female gametangia above orange-pigmented male gametangia (Fig 4B). Other strains showed varying degrees of abnormal development, but often including atypical “bloated males”, with pigment and discharge papillae like male gametangia, but not adjacent to female gametangia as in normal gametophyte development (Fig 4B). These bloated males failed to release gametes. Unusual twisted hyphae and blebbing at hyphal tips were also observed, and this abnormal phenotype strain showed a reduced growth rate (Fig 4C and 4D).

We then investigated the association of these phenotypes with presence of the virus using genome sequencing and qPCR. We sequenced the genomes of two Cali8 gametophyte cultures, one of which had the abnormal phenotype (Sub-culture 1-A) and the other of which had presumably selfed and had sporophyte morphology (Sub-culture 2-N). The abnormal Sub1-A had higher coverage of the viral region compared to the rest of the genome, though not as high of coverage as the originally sequenced strain (Fig 3D). The strain with normal sporophyte phenotype (Sub2-N) had almost no sequencing coverage of the viral region (Fig 3D), suggesting that the virus had been lost. We sequenced RNA from two additional subcultures which had distinct growth profiles (Fig 4D), displaying either diseased (Sub-culture 3-A) or normal (Sub-culture 4-N) phenotype. Surprisingly, for both subcultures the RNA-seq data show similar, very low expression of the viral region (Fig 3D). While these results are complicated, together they suggest that in the Cali8 strain the virus can be expressed though it does not consistently result in a diseased phenotype. These results also suggest that in some cases the *Allomyces* host is able to rid itself of the virus.

One mechanism that could result in virus genome disruption or elimination is meiosis. qPCR demonstrated that the gametophytes derived from different meiospores varied significantly in viral titer (ANOVA *F*-statistic = 3.36559, *p* = 0.03944) (Fig 4E). Variation is highly dynamic, and multiple subcultures of the same gametophyte produced differing amounts of virus. While qPCR of Cali8 cDNA showed that the virus is typically not expressed, MCP cDNA did amplify in one Cali8 gametophyte sub-culture, indicating expression of this viral gene. qPCR of cultures grown at high temperatures showed a greater variance in viral titer, suggesting that heat can trigger viral replication (Fig 4F). The unreliability of heat as a viral trigger indicates that other factors, such as other environmental conditions, host density, or life cycle stages, are also involved. Using both filtered cell homogenate and supernatant of a Cali8 subculture with abnormal morphology (Sub-cultures m1 and m1-3), we attempted to infect the virus-free strain Mex58 by applying these crude extracts to mating gametes but were unsuccessful.

These results strongly indicate an active infection by a giant virus in this strain of *Allomyces.* Other sequenced *Allomyces* strains had contigs similarly rich with GV hallmark genes but lacking differences in coverage from the genome-wide median estimated by read depth. It is plausible that in these strains the conditions for triggering viral release were not met under laboratory culture conditions, and thus the virus remained in a proviral state. Given the high occurrence of MCPs we suspect that additional *Allomyces* strains contain provirus EVEs that are capable of activating and forming an infectious propagule, though the triggering mechanism for viral production is unknown.

## Discussion

We report the discovery of *Mycodnaviridae*, a new family of *Nucleocytoviricota* with close phylogenetic affinities to *Algavirales* and *Imitervirales*. The three phyla of fungi in which we have found evidence of giant viruses—Blastocladiomycota, Chytridiomycota, and Zoopagomycota—have life cycle stages similar to some algae and amoebae, which may facilitate infection by viruses with persistent infection strategies, such as the phaeoviruses in *Algavirales*. In both algae and zoosporic fungi, there exist life-stages (spores and gametes) consisting of flagellated single-cells lacking a protective cell wall. The phaeoviruses have evolved stable coexistence with their hosts through a persistent viral strategy whereby these spores or gametes are infected by virus particle(s) that then integrate into the host genome [35]. Development proceeds with one copy of the virus integrated into the DNA of each cell [36]. Thalli often appear symptom-free until some unknown mechanism triggers virus emergence from the genome. Reproductive organs may develop irregularly as they become factories for viral replication [37, 38]. Very recently, another integrated viral genome was identified within a green alga and was shown to actively produce viral particles [39]. We hypothesize that fungal giant viruses in zoosporic lineages may share similar replication cycle characteristics wherein life cycle stages lacking a cell wall may be infected and then endogenized into the genome during the vegetative stages where the host proliferates with a cell wall (S5 Fig).

Genome coverage and qPCR data, transcriptional patterns, and the presence of an integrase gene in *Mycodnaviridae* are consistent with this hypothesized replication cycle. In most genomes, viral contigs have similar coverage to the host average, as would be expected for a provirus integrated into a single-celled spore/gamete that propagates the organism. Genome coverage data in one isolate of *Allomyces* (Cali8), however, suggests active replication at the time of initial sequencing. Gametophytes developed from individual meiospores of the same culture show differing amounts of MCP DNAs per qPCR, and these differences are inducible by altering the growth temperature. Though speculative, this could be indicative that, like the phaeoviruses, these reproductive structures may become GV replication factories under some conditions. Gametophyte qPCR indicated near loss of the virus in some individuals; like the phaeoviruses, it seems possible that *A. arbusculus* Cali8 mycodnavirus 1 may be lost through meiosis. Sporophytes of this isolate produce fewer MCP per qPCR compared to gametophytes or mixed cultures, consistent with endogenization at this life stage. While RNA-seq of Cali8 showed only slight expression of the viral region, this is an interesting departure from the complete lack of expression observed in Burma1F. Though we sequenced RNA at multiple life stages in Burma1F, we unfortunately did not capture RNA from the gametophyte stage, which may explain why viral activation was not observed in this isolate. The observed transcriptional patterns in Burma1F suggest that the provirus may be silenced in the host genome, also consistent with transcriptional patterns of phaeoviruses integrated in their algal hosts [40]. Further investigation in this strain may reveal conditions under which the virus is activated. Future studies should also address whether, like the phaeoviruses, the virus genome can circularize. These results highlight that viral release is complicated and may be linked with environmental and host lifecycle triggers as well as the complex life history of this fungus. These results also indicate that the host possesses mechanisms for avoiding viral release and/or restricting the spread of viral particles.

Like all cellular organisms, the evolution of the fungi has been impacted by interactions with viruses. Giant viruses are hypothesized to have originated with the diversification of eukaryotes, and metagenomic sequence data demonstrates their abundance in the environment. The lack of evidence of active infection by giant viruses in fungi before now is therefore surprising, though previous reports have shown their association [19,21,22,24,25]. Most interestingly, the EVEs reported by others are of distinct and distant relation to the clade of giant fungal viruses we report here. Phylogenetic reconstruction of the DNA polymerase B (PolB) using data from four such studies as well as *Mycodnaviridae* shows at least five unique clades of giant virus PolBs associated with Fungi (S6 Fig). Taken together, these data suggest that, like animals, fungi have historically and, perhaps, currently been hosts to multiple distinct lineages of giant viruses.

It is intriguing to consider the implications of a possible ancient association between fungi and giant viruses in relation to fungal evolution because viral gene acquisition can be an impetus for evolutionary innovation [41]. In the fungi, a transcription factor involved in cell cycle regulation that is, surprisingly, unique to fungi is hypothesized to have been acquired by the fungal ancestor through lateral gene transfer from a virus [42]. Phylogenetic evidence points to the source as either a giant virus or phage-infected bacterium. We searched MCP-containing contigs but found no homologs containing the diagnostic viral-like KilA-N domain of this cell-cycle protein, though this does not preclude giant viruses as the original source. Other evolutionary innovations resulting from giant virus association could involve novel metabolic functions. Especially in GVMAGs, a vast diversity of metabolic genes has been found [19, 43] including many involved in carbon and nitrogen metabolism. Assessing the genetic and functional impact of giant virus infection on fungal metabolism should be a future research priority. To understand the impact of the viruses, we need to resolve when and under what conditions the viruses are expressed during the life cycle.

Notably, the most contiguous viral genomes were found in *Allomyces* spp., from cultures that were recovered from filter paper stocks after 30 years of storage. This fact demonstrates desirable aspects of this fungus that bolster motivation for its revival as a model system. *Allomyces* has historically, and currently though with much less frequency, been a model for studying biological phenomena such as anisogamy [44, 45], sensorimotor systems including phototaxis and chemotaxis [46, 47], and even cellular differentiation [48]. The development of *Allomyces* as a model for studying giant viruses has enormous potential. Much of what is known about giant virus biology has been learned from protist-virus interactions or metagenomics where the host is unknown. The development of additional laboratory model systems of giant viruses and their natural hosts could advance our understanding of virus-host interactions, including immune response, viral “reprogramming” of host cells, metabolic impacts, and their ecosystem-level consequences.

The discovery of giant viruses in fungi that are restricted primarily to the zoosporic fungi inspire numerous questions that beg further investigation. How is viral expression and virion production related to the host life cycle, and vice versa? What are the metabolic impacts of infection, and how important are they on the global scale? What defense systems do fungi employ against giant viruses, and do they explain why Dikarya fungi are not infected? Giant virus model systems in zoosporic fungi may be the key to exposing these latent revelations.

## Methods

### Fungal strains, nucleic acid preparation, and Illumina sequencing

We initially searched 135 genomes in this study including 131 fungi, 92 of which are zoosporic (S1 Table). For full details about DNA preparation and sequencing of these isolates see Amses and colleagues [7]. An additional nine strains of *Allomyces* spp. were whole-genome sequenced from DNA archived in the CZEUM collection [49]. Library preparation and sequencing was performed by the Advanced Genomics Core at the University of Michigan by NovaSeq S4 300 cycle on 4% of a flowcell.

*Allomyces javanicus* California12 and *Allomyces arbusculus* Burma1F were grown in PmTG liquid media for 7–10 days at 22 ℃ with shaking at 180 rpm. Tissue was subsampled with sterile tweezers and immediately frozen and ground to a powder in liquid nitrogen. Approximately 50 mg was apportioned to 1.5mL tubes, 1 mL of TRIzol applied, and stored at −20 ℃ until further processing. For *A. arbusculus* Burma1F, the remaining tissue was decanted, rinsed once with sterile water, and replenished with sterile water. After overnight incubation at 22 ℃, tissue was decanted, rinsed once with sterile water, subsampled and processed as described above, then transferred to a sterile tea strainer and submerged in 50 mL sterile water for two hours on the benchtop. The tea strainer was removed, and the remaining liquid observed under a microscope for zoospore presence, followed by centrifugation at 4,000*g* and 4 ℃ for 12 min. The zoospore pellet was decanted, 1mL of TRIzol added, ground with a pestle, then stored at −20 ℃. The RNA extraction for all samples was completed per manufacturer protocol. Total RNAs were quality-checked on a Tapestation and had RIN values between 6.8 and 10. Samples were Poly-A enriched, and libraries were prepared and sequenced by the University of Michigan Advanced Genomics Core by NovaSeq S4 300 cycle on 1% of the flowcell.

### Genome and transcriptome assembly, annotation, and statistics

Transcriptome data were quality filtered by removing reads with Phred score <20 using Fastx-toolkit ([50]), then assembled de novo using Trinity 2.12 [51]. Hisat2 v.2.2.1 [52] was used for the RNAseq analysis: the hisat2-build function was used to build an index from genome assemblies, then RNA sequencing reads were aligned to the indexed assemblies using default parameter settings. Alignments were visualized with SeqMonk v.1.48.1 (RRID:SCR_001913).

Paired-end genomic data of *Allomyces* strains were quality-filtered by trimmomatic [53] with a sliding window of 4 bp and threshold of Phred 20, then assembled by Spades 3.15.5 [54] and annotated with both Prodigal v. 2.6.3 [55] and Augustus 3.2.1 [56] trained with *Rhizopus oryzae*.

For the genome plots of *Allomyces arbusculus* Burma 1F *and Allomyces javanicus* California12, we used Prodigal v. 2.6.3 [55] for gene prediction of soft-masked viral contigs with inputs from Funannotate v. 1.8.16 [57] which is a fungal gene annotation tool and GeneMarkS (http://exon.gatech.edu/genemark/index.html) using the viral option for input sequence type. GC skew was calculated in a 5-kb sliding window with a 1-kb step size. Circos v. 0.54 [58] was used to construct the circular genome visualization.

### Identification of viral content

We used the program ViralRecall [59] to identify putative viral genes, viral contigs, and viral regions within contigs. We first ran ViralRecall 1.0 on all assemblies from [7] (*n* = 131) with both stringent (-s 15 -m 30 -v 10) and relaxed (-s 15 -m 2 -v 2) parameters—the latter for poorer quality genome assemblies—and identified isolates containing MCP homologs with *e*-value < 1*e*^−10^. ViralRecall searches gene predictions generated using Prodigal [55]; we also performed hmmsearches (HMMER3 v3.1b2; S. [60] with the ViralRecall 2.0 GVOG hmm on a subset of our Augustus-predicted genomes. We found no differences in the number of MCPs called, and so continued characterizations for the isolates that these initial ViralRecall results predicted at least one MCP homolog. For each MCP-containing contig, we calculated GC content, intron density per the Augustus annotation, and performed BLAST searches [61] on both gene prediction datasets.

To aid in distinguishing viral genomes from within the fungal assemblies, we searched the 16 prodigal-annotated genomes previously identified to contain MCPs with HMMER3 [60] as in [19] using hmms of a subset of the nucleocytoplasmic viral orthologous genes (NCVOGs)—the 20 NCVOGs most likely to have been vertically inherited [32]. We made individual gene trees including all hits with e-values less than 1e-10 by aligning gene sequences with MAFFT version 7 using the E-INS-i algorithm [62], trimming the resulting alignments with the -automated1 method in TrimAl [63], and reconstructing trees with the approximately maximum-likelihood approach implemented in FastTree [64] with 100 bootstrap replicates, rooting at the midpoint. We also ran the latest version of ViralRecall (2.0; [59] on these 16 assemblies and used these results in combination with genome statistics to discern putatively viral contigs, which we classified as belonging to a viral genome if they met the following criteria: ViralRecall score >2, viral region length (per ViralRecall) >70% of the total contig length, and intron density >25% different from the host average*.* One isolate appeared to contain two distinct NCLDVs; we used MetaBAT2 [65] on the entire genome of *Allomyces javanicus* California12 under the default settings to separate the two NCLDV genomes.

We conducted BLASTP searches of the hybrid proteomes against nr to identify homologs (*e*-value < 1*e* − 10). Because many symbiont genes are likely misidentified as their hosts’ in the databases, we labeled a gene as “viral” or “bacterial” if any of the top hits were to a virus or bacterium. Some genes have homologs only in the fungi which we have shown also encode MCP; we labeled these “putative FNCLDV”.

To assess prevalence of fungal giant virus-like MCP throughout the fungal kingdom, we used blastp to search all unmasked fungal genomes in Mycocosm [27] using MCP of *Allomyces arbusculus* Burma1F as query (total 2,543 genomes; conducted on Dec 30, 2023). We also searched these Mycocosm genomes using the following protein models: PF04451 (Large eukaryotic DNA virus major capsid protein), IPR007542 (Major capsid protein, C-terminal), IPR031654 (Major capsid protein, N-terminal), and IPR016112 (Group II dsDNA virus coat/capsid protein) (conducted on Jan 13, 2026). The first three profile searches yielded hits only to zoosporic fungi, with the exception of a gene in *Dimargaris cristalligena* (hit to IPR007542). Genes with homology to IPR016112 were found across fungal lineages, including in Dikarya. These genes hits, if in zoosporic fungi, typically shared annotations with PF04451 and/or IPR007542; hits in non-zoosporic lineages never shared annotations with other viral or structural proteins, suggesting non-viral function. Interestingly, searching with the Major Capsid protein N-terminal (IPR031654) yielded no results, suggesting that fungal MCPs have a unique N-terminus.

### Giant virus phylogeny, orthology analysis, and KilA-N domain search

To build the giant virus species tree, the nsgtree pipeline was used (https://github.com/NeLLi-team/nsgtree) on genome representatives of the viral phylum Nucleocytoviricota [59]. In brief, 7 giant virus orthologous groups (GVOGs; [59] were identified using hmmsearch, extracted GVOGs were aligned with MAFFT (version 7.31; [62], trimmed with trimal (-gt 0.1, v1.4; [63] and concatenated. To limit the amount of missing data, we removed genomes that had less than 4 out of 7 GVOGs. A species tree was built from the supermatrix alignment using IQ-Tree (version 2.03) [66, 67] with LG + I + F + G4 and ultrafast bootstrap replications [68] and visualized in iToL [69].

To analyze the global distribution of *Mycodnaviridae*, we used the MCP as a query for a blastp using diamond (--query-cover 30 --subject-cover 30; [70] against all MCP that we previously extracted [19] from publicly available IMG metagenomes [71]. All blastp hits were then extracted and used as query for another blastp against a database consisting of all MCP extracted from representative giant virus genomes [59] and the initial *Mycodnaviridae* MCPs. We considered as bidirectional BBH the environmental MCP that had the best hit based on bitscore to the initial *Mycodnaviridae* MCPs. Geolocation of the BBHs was then used to plot the distribution of MCPs related to *Mycodnaviridae* on a world map projection.

To search for homologs of the viral KilA-N domain in *Mycodnaviridae* genomes, the source data from Fig 6 of [42] (219 protein sequences) was downloaded. Sequences were aligned using mafft (version 7.31; [62], and an hmmprofile made with HMMER hmmbuild [60], which was then used to hmmsearch the fungal giant virus genomes.

Orthofinder 2.5.2 [72] was used to identify orthologous gene clusters from the prodigal predictions of the most complete viral genomes, determined as the viral genomes containing the greatest number (at least nine) of the NCVOGs20. Visualization of the shared orthologous gene clusters was by adaptation of https://commons.wikimedia.org/wiki/File:Symmetrical_5-set_Venn_diagram.svg. Sequences of orthologous gene clusters were fed into EggNOG-mapper 5.0 [73] with default parameters but enabling SMART annotations.

### *Allomyces* capsid PCR and growth experiment

Primers were designed from an alignment of the MCP sequences of *A. javanicus* California12 and *A. arbusculus* Burma1F (F 5′ TACACCAACTTTGCGATGGA3′ R 5′ GTAGCTCGTTGACCCGACAT3′). DNA was provided by the Collection of Zoosporic Eufungi at the University of Michigan. A standard PCR was performed using GoTaq (Promega) and the products visualized by agarose gel electrophoresis for absence/presence. Burma1F was used as a positive control, and Burma3-35 served as a negative control based on absence of the gene in the sequenced genome.

We chose 6 strains that tested positive for MCP (BEA2, Burma1F, Cali8, Cali12, FijiF1, NorthCaro2), and 6 testing negative (ATCC10983, Burma3-35, CubaS7, DJ02, DJ07, Mex58) to determine phenotypic effects of the gene’s presence. Strains were grown in triplicate on Emerson’s YpSS media [74]. The inoculum for each plate was made by growing strains for 48 hours on fresh YpSS and removing a small circular inoculum by punching discs with the back of a glass pipette. After 72 hours of growth at 35 °C, linear growth was measured for each plate with two perpendicular radii. We also estimated biomass accumulation of the same strains using liquid YpSS. Triplicate 250 ml flasks with 100 ml of media were inoculated with 4 discs cut using the back of glass pipettes. The strains were grown at 35 °C for 48 hours with shaking at 120 rpm. The mycelium was harvested using miracloth filtration, transferred to pre-weighed filter papers, dried completely, and dry weight was measured. A final experiment measured thermal maxima of strains by inoculation of YpSS plates and placing them at multiple temperatures: 38, 40, 42, 44, and 46 °C. Duplicate plates were prepared from 9-day-old YpSS plates using discs excised using glass pipettes. Plates were scored for evidence of growth, where < 2 mm of growth after 1 week was considered negative.

### *Allomyces* qPCR and transmission experiments

The starting material for these experiments were resting sporangia that had been dried down onto filter paper and stored for at least 30 years when they were provided to the Fungal Genetics Stock Center by Lene Lange. Single meiospore isolates of Cali12, Burma1F, and Cali8 were obtained by placing the resting sporangia into sterile deionized water. After a period of 1–4 days, meiospores were released and plated onto YpSS medium supplemented with penicillin and streptomycin sulfate (200 mg/L). Individual germlings were found under a stereoscope and subcultured.

We estimated the amount of viral DNA in these samples by qPCR by comparison of the copy number of the MCP to actin genes in DNA extracts. Samples for qPCR were grown in ¼ strength liquid YpSS for 6–12 days at either 22 or 37 °C. DNA was extracted using a modified CTAB protocol, and extracts were quantified using a qubit fluorimeter and diluted to 0.1 ng/ul. Primers and probes were designed using PrimerQuest (IDT) (S2 Table). Probes were IDT PrimeTime probes with double quenchers. To create a standard curve we used synthetic DNA fragments (gblocks) of a known concentration. Reactions were conducted using RADIANT Lo-ROX 2× master mix (Alkali Scientific) on a QuantStudio 3 Thermal Cycler (Applied Biosystems, Thermo Fisher Scientific).

RNA was extracted from liquid and plate YPSS/4 cultures using TRIzol per manufacturer recommendations on tissue that was first ground to a powder in liquid nitrogen, then treated with DNase. cDNA was generated using LunaScript RT SuperMix Kit (NEB) with random hexamers.

We performed an experiment to test whether we could transmit the virus from Cali8 to a virus-free *Allomyces* strain (Mex58). We grew two subcultures of Cali8 that had been shown to have high viral loads (m1 and m1-3) and Burma3-35 (virus-free) in liquid Y/4 and grown for 13 days. We removed the mycelium from the media and grinded the mycelium in an eppendorf tube with a micropestle until it was homogenous. Both the homogenate and the medium were then filtered using a 0.2 µm filter and these filtrates applied to pools of Mex58 gametes which had been released from a gametophyte culture by flooding with water. We combined 0.5 ml of gametes (>10^3^) and either 0.5 ml of filtered supernatant or 0.15 ml of filtered homogenate plus 1 ml of fresh YpSS in a multiwell plate and incubated the plate for 4 days. After this time, the cells were transferred to 1 ml fresh media with a 1/10 dilution or a 1/100 dilution and grown at 22 °C for 16 days at which point a sporophyte mycelium filled the wells. There were 12 total experiments: 2 dilutions (1/10 or 1/100, 3 strains (m1, m1-3, and Burma3-35), and two inoculum types (homogenized cells or supernatant). The total mycelium of the 12 experiments was harvested, DNA extracted using a modified CTAB method, and qPCR of the MCP and actin genes used to assess whether successful transmission occurred.

### Nanopore sequencing and genome assembly

To obtain High-Molecular-Weight DNA for long-read sequencing, the strain was first grown on YpSS solid medium for 3 weeks. A block (3 mm × 3 mm) from the edge of the growing culture was then excised and immersed in DS buffer to induce zoospore release. The spore suspension was used to inoculate 100 ml fresh YpSS liquid medium, which was then incubated for 36 h at 33 °C in a floor shaker. After incubation, the cells were collected with strainer, washed once with PBS, frozen at −80 °C, and lyophilized. The frozen-dry cells were broken into powder using 4 mm glass beads and vortex at the maximum speed. Genomic DNA was then extracted using a modified CTAB protocol, and DNA quality was inspected with CHEF gel electrophoresis as previously described (https://doi.org/10.1073/pnas.2416656121).

Purified DNA was processed for library preparation and sequencing on an Oxford Nanopore MinION Mk1C device using R10 flow cells (FLO-MIN114) with MinKNOW UI (v23.07.12) following the sequencing kit guidelines. The generated Pod5 data was subjected to base calling using Dorado (v0.6.0) followed by genome assembly using Canu (v2.1.1). The resulting genome assembly was analyzed for the presence of the capsid sequence using BLASTn analysis and transposons were identified using Repeatmasker (v4.0.7; http://www.repeatmasker.org), supplemented with Dfam (v3.3), RepBaseRepeatMaskerEdition-20181026 libraries, and RepBase EMBL database (v26.04; [75, 76]. CpG methylation analysis was performed using the nanopore Modkit tool (v0.2.5-rc1).

## Supporting information

S1 Fig Phylogeny spotlighting the zoosporic fungi, adapted from [7]. Red stars indicate species found to have integrated Major Capsid Proteins. The data underlying this Figure can be found at https://github.com/Michigan-Mycology/Chytrid-Phylogenomics.
(PDF)

S2 Fig Zoosporic fungi found with Major Capsid Proteins (MCPs) demonstrated variation in genomic properties of the viral introgression. The contig containing the MCP in *Chytriomyces hyalinus* JEL345 (lower left) is a mosaic of bacterial, viral, and fungal genes, with reduced GC content and intron density compared with fungal nuclear contigs from the same genome assembly (top left). Contrarily, the MCP-containing contig in *Podochytrium* sp. JEL797 (lower right) is composed of predominantly fungal genes, with similar GC content and intron density as a fungal control contig from the same assembly (upper right). The data underlying this Figure can be found in https://doi.org/10.7302/p5ay-ng61.
(PDF)

S3 Fig Circos plot of *Allomyces javanicus* California12 mycodnavirus 1, created as for *A. arbusculus* Burma1F (see methods). The data underlying this Figure can be found in https://doi.org/10.7302/p5ay-ng61.
(PDF)

S4 Fig Left panel: Percent GC content by Coverage plots of *A. arbusculus* BEA2 (upper) and *A. arbusculus* Cali8 Illumina assemblies (lower). Contigs containing MCP genes are shown in blue, each point is sized according to its length. The contigs in Cali8 with coverage of ~3.5k were identified as viral regions per the criteria outlined in the methods, and confirmed by chromosomal placement in the Nanopore assembly; the contigs with ~2k coverage are mitochondrial. Right panel: Normalized coverage shown as a rolling average across a non-viral contig (upper) and a viral contig (lower) in three distinct Illumina assemblies of *A. arbusculus* Cali8. Cali8-parental was the originally sequenced strain. Cali8-A refers to a strain with abnormal morphology, and Cali8-N refers to a strain presenting normal morphology, as described in the text. The data underlying this Figure can be found in https://doi.org/10.7302/p5ay-ng61.
(PDF)

S5 Fig Life cycle of *Allomyces* alternates between diploid sporophyte and haploid gametophyte. (PDF)

S6 Fig Maximum-likelihood phylogeny of the DNA PolB gene including fungal and *Nucleocytoviricota* reference genes, those from *Mycodnaviridae*, and those previously ascribed to fungal EVEs, with an animal PolB outgroup. Nodes with circles have at least 70% bootstrap support. *Nucleocytoviricota* reference sequences form a well-supported clade that includes the *Mycodnaviridae* and other, distinct, clades of fungal EVEs, suggesting a rich history of association between fungi and giant viruses. The data underlying this Figure can be found in https://doi.org/10.7302/p5ay-ng61.
(PDF)

S1 Table Shown is the number of 10 key giant virus orthologous genes (GVOGs) found across fungal genomes by HMMsearch (eval < 1*e*−10). (PDF)

S2 Table Quantitative PCR primer and probe sequences. (PDF)

## Abbreviations

BBH: best blast hit
EVE: endogenous viral element
GV: giant virus
GVOG: giant virus orthologous gene
HGT: horizontal gene transfer
MCP: major capsid protein
NCVOGs: nucleocytoplasmic viral orthologous genes
