# Supplementary material for: Mycodnaviridae is a clade of giant viruses that persistently infect zoosporic fungi
Source: PLoS Biol. 2026 Aug 25;24(8):e3003937. doi: 10.1371/journal.pbio.3003937 (PMC13533422; doi:10.1371/journal.pbio.3003937)
Supplement: Supplementary file 1 [file pbio.3003937.s001.pdf]

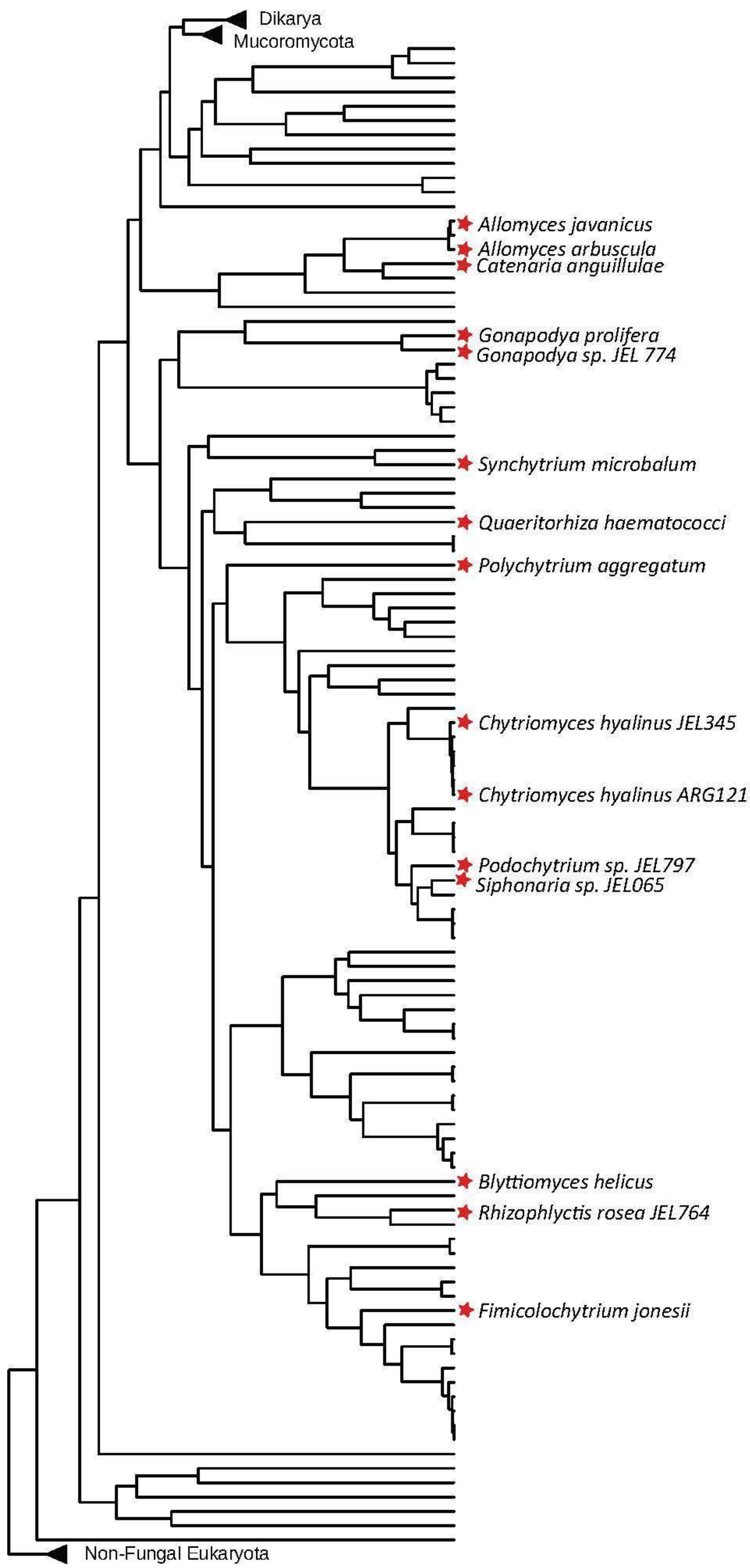

Dikarya
Mucoromycota
Allomyces javanicus
Allomyces arbuscula
Catenaria anguillulae
Gonapodya prolifera
Gonapodya sp. JEL 774
Synchytrium microbalum
Quaeritorhiza haematococci
Polychytrium aggregatum
Chytriomyces hyalinus JEL345
Chytriomyces hyalinus ARG121
Podochytrium sp. JEL797
Siphonaria sp. JEL065
Blyttiomyces helicus
Rhizophlyctis rosea JEL764
Fimicolochytrium jonesii
Non-Fungal Eukaryota
