# Supplementary material for: Mycodnaviridae is a clade of giant viruses that persistently infect zoosporic fungi
Source: PLoS Biol. 2026 Aug 25;24(8):e3003937. doi: 10.1371/journal.pbio.3003937 (PMC13533422; doi:10.1371/journal.pbio.3003937)
Supplement: Supplementary file 2 [file pbio.3003937.s002.pdf]

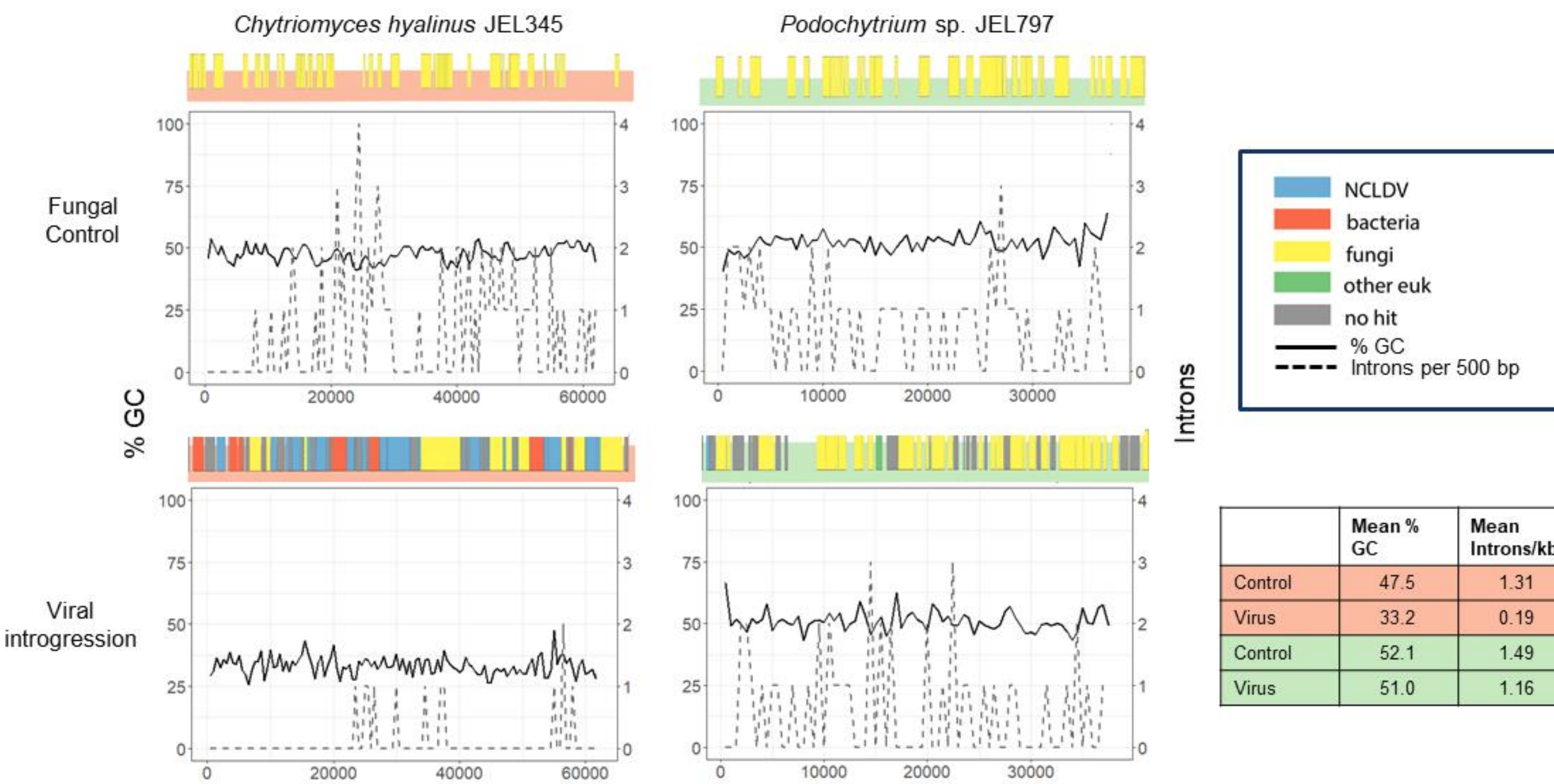

	Mean % GC	Mean Introns/kb
Control	47.5	1.31
Virus	33.2	0.19
Control	52.1	1.49
Virus	51.0	1.16
