# Supplementary material for: Mycodnaviridae is a clade of giant viruses that persistently infect zoosporic fungi
Source: PLoS Biol. 2026 Aug 25;24(8):e3003937. doi: 10.1371/journal.pbio.3003937 (PMC13533422; doi:10.1371/journal.pbio.3003937)
Supplement: Supplementary file 3 [file pbio.3003937.s003.pdf]

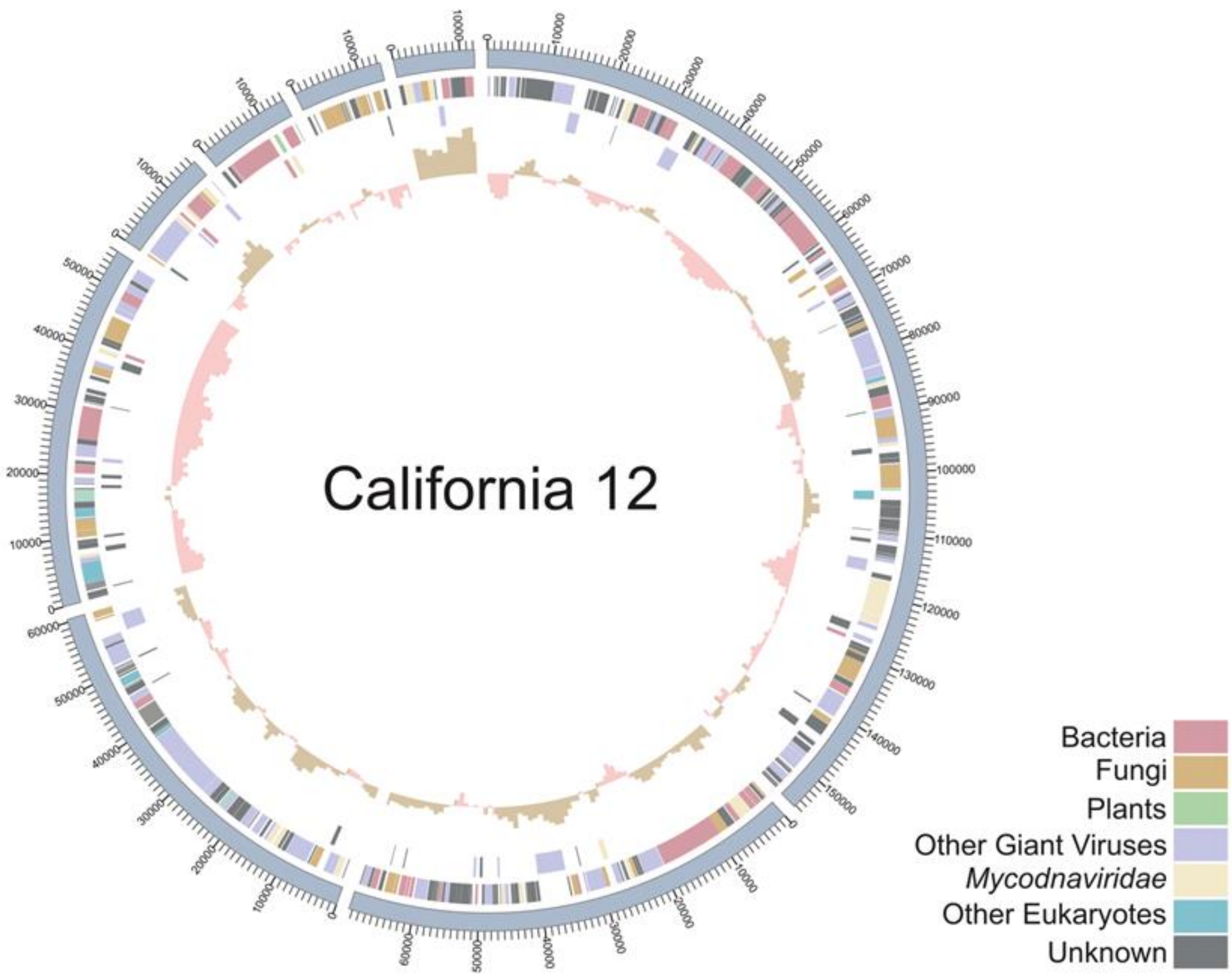
California 12
Bacteria
Fungi
Plants
Other Giant Viruses
Mycodnaviridae
Other Eukaryotes
Unknown
