# Supplementary material for: Mycodnaviridae is a clade of giant viruses that persistently infect zoosporic fungi
Source: PLoS Biol. 2026 Aug 25;24(8):e3003937. doi: 10.1371/journal.pbio.3003937 (PMC13533422; doi:10.1371/journal.pbio.3003937)
Supplement: Supplementary file 4 [file pbio.3003937.s004.pdf]

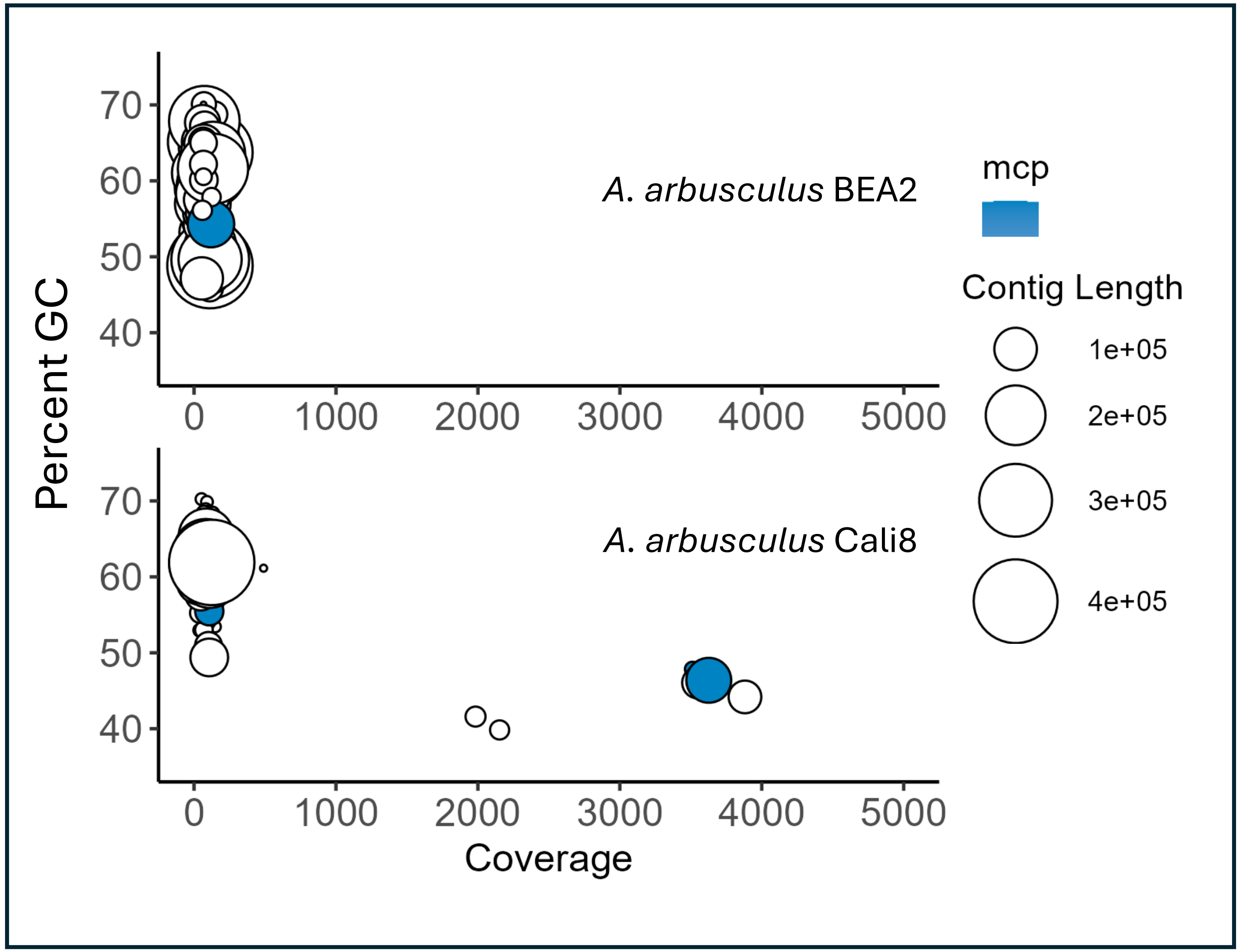
Percent GC
70
60
50
40
0
1000
2000
3000
4000
5000
A. arbusculus BEA2
A. arbusculus Cali8
Coverage
mcp
Contig Length
1e+05
2e+05
3e+05
4e+05

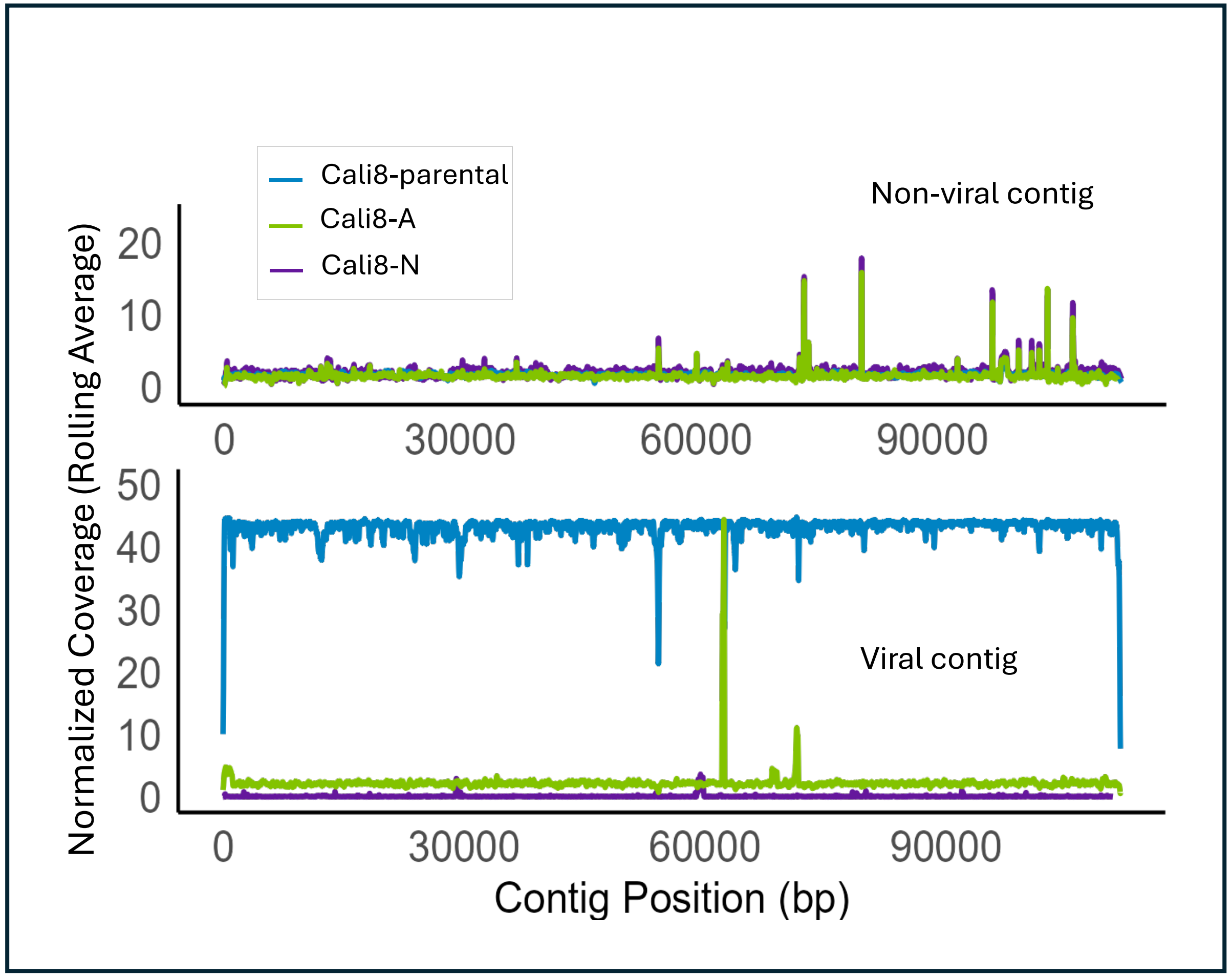
Cali8-parental
Cali8-A
Cali8-N
Non-viral contig
Viral contig
Normalized Coverage (Rolling Average)
20
10
0
50
40
30
0
30000
60000
90000
Contig Position (bp)
