# Supplementary material for: Mycodnaviridae is a clade of giant viruses that persistently infect zoosporic fungi
Source: PLoS Biol. 2026 Aug 25;24(8):e3003937. doi: 10.1371/journal.pbio.3003937 (PMC13533422; doi:10.1371/journal.pbio.3003937)
Supplement: Supplementary file 5 [file pbio.3003937.s005.pdf]

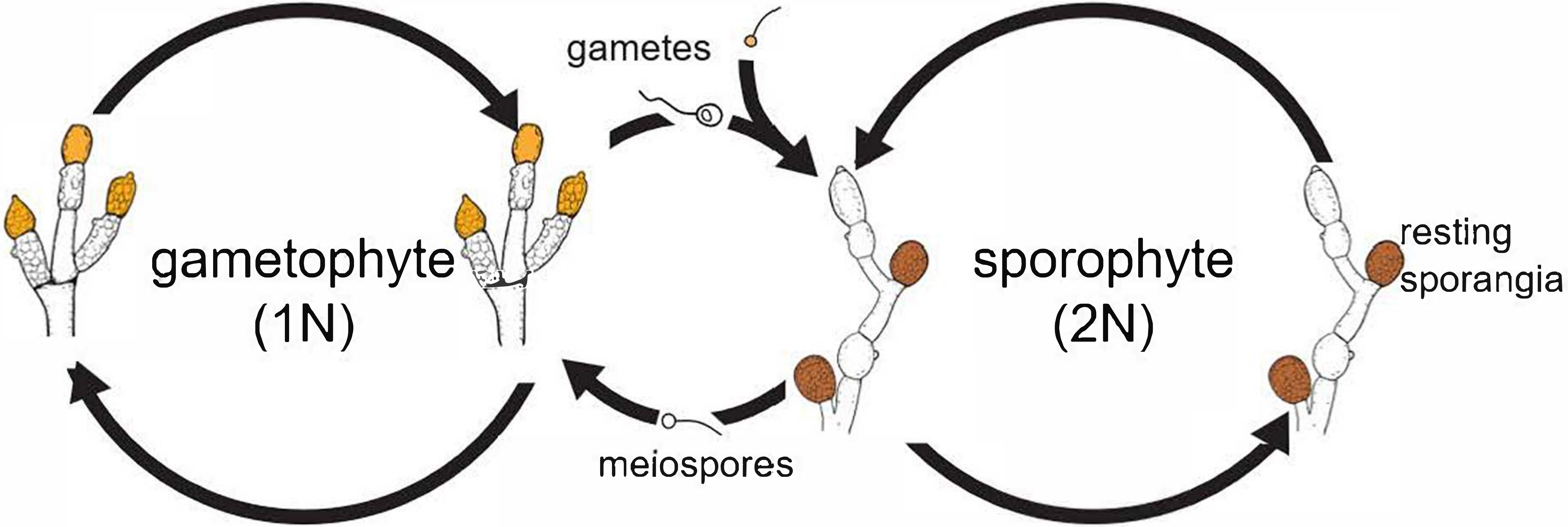

gametes
gametophyte
(1N)
sporophyte
(2N)
resting
sporangia
meiospores
