# Supplementary material for: Mycodnaviridae is a clade of giant viruses that persistently infect zoosporic fungi
Source: PLoS Biol. 2026 Aug 25;24(8):e3003937. doi: 10.1371/journal.pbio.3003937 (PMC13533422; doi:10.1371/journal.pbio.3003937)
Supplement: Supplementary file 6 [file pbio.3003937.s006.pdf]

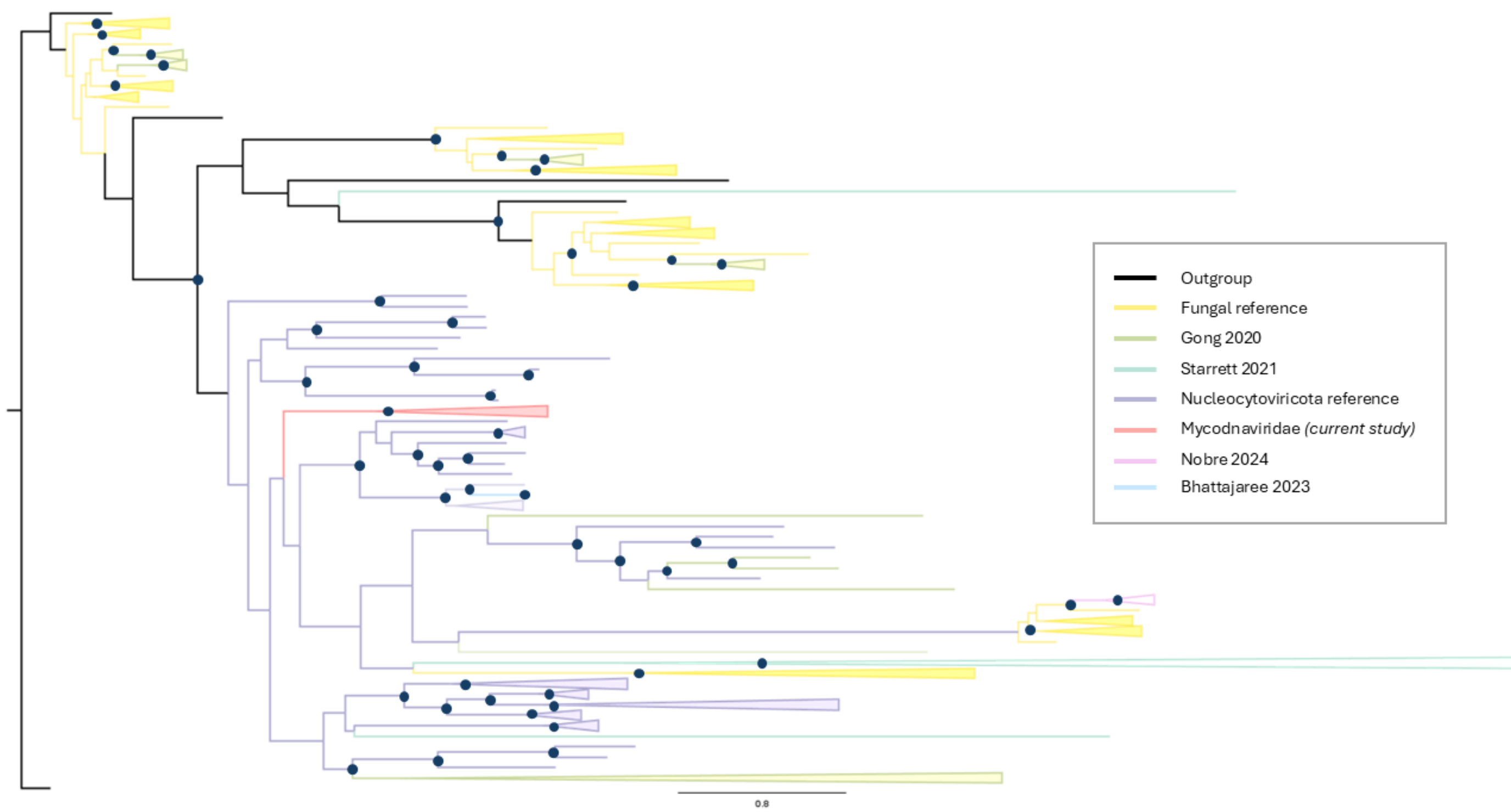

Outgroup
Fungal reference
Gong 2020
Starrett 2021
Nucleocytoviricota reference
Mycodnaviridae *(current study)*
Nobre 2024
Bhattajaree 2023
0.8
