# Supplementary material for: Mycodnaviridae is a clade of giant viruses that persistently infect zoosporic fungi
Source: PLoS Biol. 2026 Aug 25;24(8):e3003937. doi: 10.1371/journal.pbio.3003937 (PMC13533422; doi:10.1371/journal.pbio.3003937)
Supplement: Supplementary file 7 [file pbio.3003937.s007.pdf]

Isolate	Phylum	MCP	VLTF3	D5	A32	SFII	mRNAc	RNR	RNAPS	RNAPL	PolB
Arthrobotrys_oligospora_ATCC_24927	Ascomycota	0	0	0	0	0	0	0	8	8	4
Candida_arabinofermentans_NRRL_YB-2248		0	0	0	0	2	2	1	3	3	4
Hortaea_werneckii_EXF-2000		0	0	0	0	4	4	2	6	10	10
Neurospora_crassa_OR74A		0	0	0	0	1	2	1	2	4	6
Pecoramyces_ruminatium_C1A		0	0	4	0	0	1	0	11	7	5
Saccharomyces_cerevisiae_S288C		0	0	0	0	2	2	2	3	3	4
Saccharomycopsis_capsularis_NRRL_Y-17638		0	0	0	0	2	2	2	3	3	4
Schizosaccharomyces_pombe_972h		0	0	0	0	1	2	2	3	3	4
Yarrow_lipolytica_CLIB122		0	0	0	0	2	2	1	3	3	4
Armillaria_ostoyae_C18_9	Basidiomycota	0	0	0	0	0	0	23	8	7	2
Coprinopsis_cinerea_okayama7_130		0	0	0	0	0	1	2	4	3	3
Mixia_osmundae		0	0	0	0	0	0	4	4	12	4
Puccinia_graminis_f._sp._tritici_CRL_75-36-700-3		0	0	0	0	0	0	0	7	9	3
Ustilago_maydis_521		0	0	0	0	2	2	1	4	3	4
Backusella_circina_FSU_941	Mucoromycota	0	0	0	0	1	0	3	7	8	5
Bifiguratus_adelaidae_AZ0501		0	0	1	0	0	1	1	11	8	7
Gigaspora_rosea_DAOM_194757		0	0	0	0	0	0	3	7	7	77
Hesseltinella_vesiculosa_NRRL_3301		0	0	0	0	1	0	1	8	8	3
Lichtheimia_corymbifera_FSU_9682		0	0	0	0	1	0	4	7	10	2
Lobosporangium_transversale_NRRL_3116		0	0	0	0	1	0	2	12	10	6
Mortierella_elongata_AG-77		0	0	1	0	1	1	3	10	9	7
Mucor_circinelloides_f._circinelloides_1006PhL		0	0	0	0	1	0	3	7	5	6
Phycomyces_blakesleeanus_NRRL_1555		0	0	0	0	1	0	1	6	9	5
Rhizophagus_irregularis_DAOM_181602		0	0	0	0	0	0	2	12	9	44
Rhizopus_delemar_RA_99-880		0	0	0	0	1	0	2	14	11	5
Rhizopus_microsporus_var._microsporus_ATCC_52814		0	0	0	0	1	0	1	5	8	4
Saksenaea_vasiformis_B4078.G233		0	0	0	0	1	0	2	7	8	5
Syncephalastrum_racemosum_NRRL_2496		0	0	0	0	1	1	2	11	8	5
Umbelopsis_ramanniana_AG		0	0	0	0	1	0	2	8	9	4
Basidiobolus_heterosporus_B8920.N168	Zoopagomycota	0	0	3	0	0	0	3	12	10	5
Basidiobolus_meristosporus_CBS_931.73		0	0	0	0	1	0	3	9	11	4
Coemansia_reversa_NRRL_1564		0	0	0	0	2	2	1	4	6	4
Conidiobolus_coronatus_NRRL_28638		0	0	1	0	0	0	1	5	4	6
Conidiobolus_thromboides_FSU_785		0	0	2	0	1	2	1	9	7	3
Linderina_pennispora_ATCC_12442		0	0	0	0	3	2	1	9	10	6
Martensiomyces_pterosporus_CBS_209.56		0	0	0	0	2	1	1	3	4	4
Piptocephalis_cylindrospora_RSA_2659		0	0	0	0	0	1	0	0	3	1
Ramicandelaber_brevisporus_CBS_109374		0	0	0	0	2	2	1	5	5	5
Syncephalis_fuscata_S228		0	0	0	0	0	0	1	11	5	0
**Allomyces_arbuscula_Burma_1F**	Blastocladiomycota	**2**	**1**	**2**	**2**	**1**	**1**	**2**	**4**	**11**	**6**
**Allomyces_javanicus_California_12**		**5**	**2**	**9**	**4**	**3**	**2**	**4**	**4**	**11**	**9**
Allomyces_macrogynus_ATCC_38327		0	0	2	9	0	0	2	7	14	9
Blastocladiella_britannica		0	0	0	0	0	0	2	4	6	3
**Catenaria_anguillulae_PL171_v2.0**		**1**	**0**	**1**	**0**	**1**	**1**	**5**	**8**	**12**	**8**
Coelomomyces_lativittatus_CIRM-AVA-1-Meiospore		0	0	0	0	0	0	2	5	4	4
Paraphysoderma_sedebokerense_JEL0847		0	0	5	3	1	2	3	10	12	9
Anaeromyces_robustus		0	0	4	0	0	4	0	8	8	6
Batrachochytrium_dendrobatidis_JAM81		0	0	0	0	0	0	2	5	6	2
Batrachochytrium_dendrobatidis_JEL423		0	0	0	0	0	0	2	5	6	2
Batrachochytrium_salamandrivorans		0	0	0	0	0	0	2	6	5	2
**Blyttiomyces_helicus_single-cell_v1.0**		**8**	**2**	**29**	**2**	**2**	**3**	**7**	**2**	**16**	**9**
Blyttiomyces_sp._JEL0837		0	0	0	0	0	0	0	0	0	0
Boothiomyces_macroporosum_PLAUS21		0	0	1	0	0	0	3	10	21	6
Borealophylctis_nickersoniae_WJD170		0	0	36	1	0	0	1	11	6	2
Caulochytrium_protostelioides_ATCC_52028		0	0	0	0	2	2	1	3	3	4
Chytridium_lagenaria_Arg66		0	0	0	0	0	0	1	9	6	0

Isolate	Phylum	MCP	VLTF3	D5	A32	SFII	mRNAc	RNR	RNAPS	RNAPL	PolB
Chytriomyces_confervae_CBS_675.73	Chytridiomycota	0	0	2	0	0	1	1	13	11	1
Chytriomyces_hyalinus_ARG085		0	0	0	0	0	0	1	13	9	1
**Chytriomyces_hyalinus_ARG121**		**1**	**0**	**5**	**0**	**0**	**0**	**2**	**8**	**12**	**1**
Chytriomyces_hyalinus_JEL0117		0	0	5	0	0	0	3	14	14	0
Chytriomyces_hyalinus_JEL0176		0	0	3	0	0	1	2	14	9	1
**Chytriomyces_hyalinus_JEL0345**		**2**	**1**	**46**	**3**	**1**	**2**	**4**	**8**	**17**	**5**
Chytriomyces_sp._MP_71		0	0	0	0	0	0	2	15	11	5
Cladochytrium_replicatum_JEL714		0	0	0	0	0	1	2	12	11	1
Cladochytrium_tenue_CCIBt4013		0	0	0	0	0	0	4	13	13	2
Clydaea_vesicula_JEL0476		0	0	5	0	9	2	2	11	8	5
Dinochytrium_kinnereticum_KLL_TL_06062013		0	0	0	0	0	0	1	4	9	1
Entophlyctis_helioformis_JEL805		0	0	0	0	0	0	1	16	10	4
Entophlyctis_luteolus_JEL0129		0	0	1	0	0	0	4	10	15	2
Entophlyctis_sp._JEL0112		0	0	1	0	0	0	4	12	15	2
**Fimicolochytrium_jonesii_JEL569**		**1**	**0**	**23**	**0**	**3**	**2**	**3**	**11**	**6**	**3**
**Gaertneriomyces_semiglobifer_Barr_43**		**0**	**0**	**19**	**1**	**2**	**1**	**4**	**9**	**6**	**4**
Gaertneriomyces_sp._JEL0708		0	0	4	0	2	2	6	10	8	7
Geranomyces_michiganensis_JEL0563		0	0	4	0	1	1	2	8	10	5
Geranomyces_variabilis_JEL0379		0	0	4	0	1	2	2	9	10	5
Geranomyces_variabilis_JEL0389		0	0	7	0	1	1	1	8	9	4
Geranomyces_variabilis_JEL0566		0	0	5	0	1	1	1	8	9	4
Geranomyces_variabilis_JEL0567		0	0	4	0	1	1	2	9	12	4
Geranomyces_variabilis_JEL559		0	0	4	0	1	1	2	8	12	4
Globomyces_pollinis-pini_Arg68		0	0	0	0	0	0	1	9	9	1
**Gonapodya_prolifera**		**2**	**4**	**7**	**1**	**1**	**1**	**0**	**7**	**13**	**5**
**Gonapodya_sp._JEL0774**		**5**	**1**	**28**	**1**	**1**	**1**	**2**	**7**	**16**	**3**
Gorgonomyces_haynaldii_MP57		0	0	0	0	1	1	2	8	6	5
Homolaphlyctis_polyrhiza_JEL142		0	0	0	0	0	1	1	9	8	4
Hyaloraphidium_curvatum_SAG235-1		0	0	6	2	0	0	1	6	9	4
Irineochytrium_annulatum_JEL0729		0	0	0	0	3	0	5	10	9	4
Kappamyces_sp._JEL0680		0	0	0	0	0	2	2	8	9	5
Lobulomyces_angularis_JEL0522		0	0	2	0	0	1	2	10	9	4
Lobulomyces_sp._JEL0476		0	0	5	0	0	1	2	11	8	6
Neocallimastix_californiae_G1		0	0	23	0	0	13	0	46	70	35
Nowakowskiella_sp._JEL0407		0	0	0	0	0	0	2	26	10	0
Obelidium_mucronatum_JEL802		0	0	0	0	0	0	4	8	18	1
Olpidium_bornovanus_UCB_F19785		0	0	1	0	0	1	1	15	16	4
Olpidium_sp._PSC023		0	0	1	0	2	0	1	5	4	3
Paranamyces_uniporus_JEL0695		0	0	0	0	1	1	3	19	17	7
Phlyctochytrium_bullatum_JEL0754		0	0	1	0	0	0	2	11	10	1
Phlyctochytrium_planicorne_JEL0388		0	0	0	0	0	0	0	9	8	1
Physocladia_obscura_JEL0513		0	0	6	0	0	1	2	8	10	1
Piromyces_finnis		0	0	7	1	0	3	0	16	22	7
Piromyces_sp._E2		0	0	2	0	0	0	0	9	10	7
**Podochytrium_sp._JEL0797**		**1**	**1**	**1**	**0**	**0**	**0**	**2**	**6**	**9**	**1**
**Polychytrium_aggregatum_JEL109**		**3**	**1**	**27**	**1**	**0**	**0**	**9**	**14**	**14**	**4**
Powellomyces_hirtus_BR81		0	0	4	0	1	1	5	8	8	4
Powellomyces_hirtus_CBS809.83		0	0	12	0	2	1	0	9	7	4
**Quaeritorhiza_haematococci_JEL0916**		**8**	**1**	**85**	**0**	**2**	**2**	**3**	**12**	**9**	**3**
Rhizoclosmatium_globosum_JEL800		0	0	6	0	0	0	2	13	22	1
Rhizoclosmatium_hyalinum_JEL0917		0	0	2	0	0	0	2	12	15	3
Rhizophlyctis_rosea_JEL0318		0	0	35	1	0	0	4	12	6	1
**Rhizophlyctis_rosea_JEL0764**		**1**	**0**	**27**	**2**	**1**	**0**	**3**	**10**	**7**	**1**
Rhizophydium_sp._JEL0728		0	0	0	2	0	0	5	15	23	6
Rhizophydium_sp._JEL0801		0	0	0	0	0	0	1	12	11	3
Rhizophydium_sp._JEL0829		0	0	1	0	0	2	2	11	11	6

Isolate	Phylum	MCP	VLTF3	D5	A32	SFII	mRNAc	RNR	RNAPS	RNAPL	PolB
Rhizophydium_sp._JEL0838		0	0	2	5	1	0	3	7	11	6
Rhizophydium_sp._JEL0842		0	0	0	0	0	0	2	6	9	1
Rhizophydium_sp._JEL0866		0	0	6	3	1	3	3	9	13	9
**Siphonaria_sp._JEL0065**		**3**	**0**	**5**	**0**	**0**	**1**	**3**	**20**	**25**	**7**
Spizellomyces_palustris_CBS455.65		0	0	2	0	1	2	3	8	7	4
Spizellomyces_palustris_CBS455.65		0	0	2	0	1	2	3	8	7	4
Spizellomyces_punctatus_DAOM_BR117		0	0	5	0	1	1	2	10	8	4
Synchytrium_endobioticum_MB42		0	0	0	0	0	0	1	7	7	2
**Synchytrium_microbalum_JEL0517**		**3**	**1**	**13**	**1**	**1**	**1**	**3**	**6**	**4**	**1**
Thoreauomyces_humboldtii_JEL0095		0	0	22	1	1	1	2	9	6	3
Triparticalcar_arcticum_BR59		0	0	37	0	1	1	2	4	5	3
Unknown_unknown_JEL0888		0	0	0	0	0	0	2	4	6	5
Zopfochytrium_polystomum_WB228		0	1	45	0	0	1	2	9	11	6
Paramicrosporidium_saccamoebae_KSL3	Cryptomycota	0	0	0	0	2	2	1	3	4	3
Rozella_allomycis_CSF55		0	0	0	0	1	0	1	8	9	4
Rozella_multimorpha		0	0	0	0	0	1	1	10	10	4
Rozella_sp._PSC023		0	0	0	0	1	0	2	4	5	6
Mitosporidium_daphniae_UGP3		0	0	0	0	0	0	1	7	10	5
Fonticula_alba_ATCC_38817	Outgroup	0	0	0	0	0	1	1	7	11	4
Capsaspora_owczarzaki_ATCC_30864		0	0	3	0	2	2	3	2	4	5
Drosophila_melanogaster		0	0	0	0	0	1	1	4	9	2
Paraphelidium_tribonemae_X-108		0	0	5	1	11	3	11	25	35	7
Monosiga_brevicolis_MX1		0	0	0	1	1	0	1	5	5	3
