# Supplementary material for: Mycodnaviridae is a clade of giant viruses that persistently infect zoosporic fungi
Source: PLoS Biol. 2026 Aug 25;24(8):e3003937. doi: 10.1371/journal.pbio.3003937 (PMC13533422; doi:10.1371/journal.pbio.3003937)
Supplement: Supplementary file 8 [file pbio.3003937.s008.pdf]

Primer/Probe	Sequence
Cali8_cap_44-F	5’- CCCTCTATGTCCCTCTCATCTT
Cali8_cap_44-R	5’- CGGAACTGGCGGAATGTAAT
Cali8_cap_probe	5’- ATTGCCCTCCTCCACCACGA
Cali8_cap_gblock	5’CAACCCTCTATGTCCCTCTCATCTTCTGGTTCAACCGCAACCCTGGCCTCGCCCTTCCCCTCATTGCCCTCCTCCACCACGACGTCAAAATTAACATTACATTCCGCCAGTTCCGCGACTGCTATGTGCA
Cali8_actin_4206-F	5’- GTAGCAGAGCTTCTCCTTGATG
Cali8_actin_4206-R	5’- GCACTGGCTGCAGAAGAT
Cali8_actin_4206_probe	5’- ACGATTTCGAGCTCGGCCGT
Cali8_actin_4206_gblock	5’TCGAGCGCAACGTAGCAGAGCTTCTCCTTGATGTCGCGCACGATTTCGAGCTCGGCCGTGGTCGTGAGCGAGTGGCCGCGCTCCATGAGGATCTTCTGCAGCCAGTGCGTCAGGTCGCGACCGGCGAGGTC
Cali8_actin PRB Set 1	5’-HEX /ACGATTTCG /ZEN /AGCTCGGCCGT /3’ IABkFQ/
Cali8_capsid PRB Set 1	5’-6-FAM /ATTGCCCTC /ZEN /CTCCACCACGA /3’ IABkFQ/
