# Supplementary material for: Mycodnaviridae is a clade of giant viruses that persistently infect zoosporic fungi
Source: PLoS Biol. 2026 Aug 25;24(8):e3003937. doi: 10.1371/journal.pbio.3003937 (PMC13533422; doi:10.1371/journal.pbio.3003937)
Supplement: Supplementary file 9 [file pbio.3003937.s009.pdf]

“Discovery of *Mycodnaviridae*, a clade of giant viruses that persistently infect zoosporic fungi”

Jillian M. Myers, Frederik Schulz, Saleh Rahimlou, Vikas Yadav, Sheng Sun, Kevin R. Amses, D. Rabern Simmons, Michelle Orozco-Quime, Joseph Heitman, Jason E. Stajich, Timothy Y. James

Dear Reviewers,

We are grateful for your time reading our manuscript “Discovery of giant viruses and past and present infections in zoosporic fungi” and for your very helpful comments, experimental suggestions, and identification of weaknesses and areas needing clarity. We have worked to address each point of concern and believe the paper to be greatly improved as a result. ***We sincerely thank you for your role in this improvement.*** Please see below for details of how we addressed each concern. Note that all line numbers refer to the revised version.

Reviewer 1:

Viruses are among the terra incognita in biology. Research into viruses in many eukaryotic lineages has only recently been accelerated by rapid advances in (meta)genomics. In the fungal kingdom, research into viruses has been lacking behind other eukaryotic lineages, and DNA and RNA viruses have only recently been detected, underscoring the tremendous knowledge gaps surrounding viruses in fungi.

The short report 'Discovery of giant viruses as past and present infections of zoosporic fungi' by Myers and colleagues report on genomics, phylogenetics, and microscopic research to detect and characterize giant viruses in zoosporic fungi. The manuscript aims to address a timely and interesting research question, and the general approach (experimental design) is reasonable; all data needed to replicate the work is present in the public domain or has been deposited by the authors. However, I have several concerns and suggestions on the performed analyses, the robustness of the results, and the clarity of the manuscript that need to be addressed.

Comment:

The bioinformatics analyses need clarification and reanalyses:

- *it is not clear why the manuscript first reports MCP in a small subset of fungi and only later queries a bigger dataset (Mycocosm; L69ff). It seems more logical and in line with the aim of the article if the authors would have queried the more comprehensive dataset from the start.*

> **Response:**
> Our findings of giant virus major capsid proteins in zoosporic fungi was a serendipitous one, as we mention in the first paragraph (L51-53). Some of the authors previously participated in a large genomics study of these fungal lineages, as they are a particularly understudied group, and in that process we stumbled upon an MCP in *Allomyces*. As we worked to decipher whether this was an isolated instance of an endogenous viral element or a signal of something more widespread, we explored whether MCPs were in other, closely related, lineages. As we describe, we found them scattered throughout the

Chytridiomycota and Blastocladiomycota. Our previous work demonstrated novel RNA virus diversity in these understudied fungal groups, and we suspected these MCPs might be restricted. To test this idea, we then expanded our searches throughout the fungal kingdom.

We could explain the science in a number of ways, but we chose to divulge that what turned out to be a rather interesting finding was essentially a happy accident. This choice of narrative is honest and, we hope, exemplifies something important about the process of discovery. To address this and other reviewer comments we have made substantial modifications to the introduction which we hope better articulates our story.

Comment:

- *MCP homologs are initially identified using HMMer searches but Mycocosm as well as the metagenomic data is queried via (bidirectional) blast. To detect remote homology and capture the breadth of these viruses throughout fungi, the latter searches need to be repeated via more sensitive HMMer searches and/or via profile-vs-profile searches.*

Response:

Using HMMs to search all of the nearly 4,000 fungal genomes in Mycocosm would be ideal to detect remote homology of GV throughout the kingdom and a future study should undertake this. For the goals of this study, we deemed this more computationally intensive than was within our scope. However, we extended our query by using protein profile searches of the Mycocosm. We used the protein profiles for "Capsid_NCLDV" PFam (PF04451) and Interpro capsid domains IPR007542, IPR031654, and IPR016112 (L96-98, methods on L708-718) to perform a new search against Mycosm. Since we are basing our detection on the presence of a capsid and these profile searches yielded similar results, this request should be satisfied.

Comment:

- *it is not clear from the manuscript if all genomes and their contigs are scanned for signals of viral contigs with ViralRecall. It is also unclear how the size of the viruses is determined; is it the entire size of a contig? This could be problematic given the chimeric origin of the genes reported on these contig, suggesting that these viral sequences rapidly change. Lastly, algorithms to detect viral contigs differ significantly (see e.g., Wu et al. 2024). How would the application of different algorithms to classify viral contigs impact the manuscript's results?*

Response:

We have clarified in the methods that we ran ViralRecall on the original fungal assemblies (n= 131) sequenced in Amses 2022 (L672-673).

Determining the size of the virus is, indeed, challenging and there is no perfect method to determine size in lieu of viral particle isolation. We report our assemblies cautiously as genome size estimations, which aligns with other reports of GV genome assembly sizes

from genomic and metagenomic datasets. We achieved these estimates as reported in the methods (L692-696):

> "We also ran the latest version of ViralRecall (2.0; Aylward and Moniruzzaman 2021) on these 16 assemblies and used these results in combination with genome statistics to discern putatively viral contigs, which we classified as belonging to a viral genome if they met the following criteria: ViralRecall score >2, viral region length (per ViralRecall) >70% of the total contig length, and intron density > 25% different from the host average."

Later in the manuscript we describe results of the long-read sequencing of two isolates, which confirmed that the methods above resulted in the identification of viral contigs that are indeed side-by-side on a chromosome. Methylation patterns and read pileups from both short-read and RNA sequencing provide additional evidence that our methods resulted in the identification of contigs that interact with host machinery differently than the rest of the host DNA.

In regards to the use of different algorithms to classify viral contigs, we selected ViralRecall because it is, to our knowledge, the only algorithm targeting giant viruses specifically and it is largely based on the very sensitive profile hmm searches of giant virus genes. As described above, we did not actually use the ViralRecall results alone to classify viral contigs.

We thank the reviewer for recommending Wu *et al* 2024. In this article, the authors discuss previous studies comparing the performance of various bioinformatic virus identification tools. They cite Pratama *et al* 2021, which "found that viral identification efficiency increases with fragment length, and almost all tools can correctly identify viral contigs of 10kb or longer." Wu *et al* further discuss that gene content-based tools "maximize the true positive rate and minimize the false positive rate at length >3kb" compared to K-mer and BLAST-based tools. Based on those assessments, there should be suitable confidence in our results since ViralRecall is gene content-based and viral contigs that met our criteria were >10kb, with the exception of one contig that was >4kb.

Comment:
In L105, tetramer frequency is used to bin contigs. Are the binning results also supported by read coverage, e.g., by mapping the original sequencing data back to viral bins?

Response:
The read coverage pattern for this isolate, *Allomyces javanicus* Cali12, reflects that observed for isolate *A. arbusculus* B1F whereby the viral and fungal regions are similar. This suggests that, at the time measured, the virus was endogenous without active infection.

Comment:
It is mentioned in the manuscript that MCP in fungal genomes are indications of ancient viral association / infections, which then would also explain the mixed origins of genes associated with the viruses as well as differences in GC content etc. It is shown, but not further discussed, that MCP and viral contigs have less introns that the average fungus. Is it known if fungal viruses have introns? The presence of some introns in viral sequences might provide additional evidence to when viruses entered the hosts.

> **Response:**
> The presence of introns in fungal virus genomes has not been well explored. The genomes of previously identified fungal viruses are overwhelmingly comprised of RNA which, generally, only rarely include introns. This is the first report of a dsDNA fungal virus, and the few reported ssDNA fungal viruses have tiny genomes such that they are unlikely to include introns.
>
> We use introns predicted by Augustus as a character to distinguish between viral and host regions. However, we do not know if the intronic regions predicted by Augustus in the viral contigs function as such. When Augustus is used to annotate reference giant virus genomes (in *Imitervirales* and *Algavirales*), introns are also predicted and at similar density to those found in *Mycodnaviridae.* To our knowledge, it is also currently unknown whether these predicted intronic regions in reference GVs function as such. Nevertheless, it proves a useful character.

Comment:
Figure 3B shows the read coverage of contig3 with viral sequence embedded. The coverage at the virus seems to be less than the remainder of the contig. Could the authors please provide an explanation for this observation?

> **Response:**
> This is indeed a curious observation. Perhaps the fungus has a defensive mechanism for excising the virus and destroying it. Our new qPCR data also suggests that the virus may be lost through meiosis. Those sequencing data came from DNA of a mixed culture of sporophytes, gametophytes, and resting sporangia. We believe it likely that this represents a heterogeneous mixture of haploid and diploid cells, some of which have lost the virus. However, much remains to be understood.

Comment:
While a short report is surely a suitable venue for these observations, the rationale for some experiments is currently very challenging to follow and details to contextualize the results are missing. To enhance clarity, the results and discussion section should be merged, additional background needs to be added (see also comments below), the motivation for individual experiments (e.g., L77) need to be better explained, and solid results need to be clearer separated from hypothesis or assumptions, e.g., in the abstract, L237, or L183; the presented

data shows that the virus is not expressed but does not provide evidence that the host actively silences the virus.

Response:
Thank you for pointing out these weaknesses and for your suggested improvements. We have made substantial changes to the work to comprehensively address these concerns:

- Necessary background on GV added to the introduction (L54-75)
- Some of the more obscure experiments were removed, and replaced with new experiments that better address the questions posed regarding viral activation and expression (new results section starts L253)
- Language regarding "active silencing" of the virus has been removed.
- A life cycle of this interesting species was more clearly described (L259-266), and a supplemental figure added (Fig S5), which is important for understanding the context of the phenotypic results and the new qPCR experiments.

Comment:
L57: it is not clear what fungal homologs have been identified in viral metagenomes. Please clarify this statement.

Response:
There have been many (over 100) putative fungal homologs detected in metagenomics-assembled giant viruses per the references supplied in-text (L81-82, Schulz et al. 2018, Schulz et al. 2020, Bhattacharjee et al. 2023), including a ubiquitin-conjugating enzyme, regulatory protein MIG1, peptidase_M24, DEAD-like helicase C, transketolase, *etc*. As the number and variety is so expansive, we decided to continue to point the reader to the references rather than expand further in this text. For the reviewer's detailed interest, we point their attention particularly to Suppl. Table 6 in Schulz 2020.

Comment:
L60: please provide more details how the evidence differs, and what the authors refer to as 'robustness'

Response:
We have edited this section for clarity, expanded on the differences, and have removed the word "robustness". The expansion now reads "Previous studies have suggested associations between *Nucleocytoviricota* and fungi through the identification of putative fungal homologs in metagenomic-assembled giant viruses (Schulz et al. 2018, Schulz et al. 2020, Bhattacharjee et al. 2023) and giant virus EVEs in fungal genomes (Gallot-Lavallée and Blanc 2017, Gong et al. 2020, Zhao et al. 2023, Nobre 2024). Of these mentioned, only the EVE studies directly connect fungi with viruses, while the metagenomics studies predict fungal affiliation of the viruses based on gene homology

and, in the case of Bhattacharjee et al. 2023, the dominance of fungi in the environment. The latter does not preclude the possibility of non-fungal hosts."

Comment:
L91: the implication of this observation remains unclear and needs to be better explained.

Response:
Polintoviruses are at the "root of eukaryotic dsDNA virus, transposon, and plasmid evolution" (Koonin 2015). Our finding of a new clade of eukaryotic virus with type B DNA polymerase and other GVOGS as well as a polinton-like integrase could help flesh out the evolutionary relationships of these groups. We have added this contextualizing language on L117-120.

Comment:
L112: what is the authors' evidence that the two viruses are derived from the parents. Could these not be merely the result of a co-infection?

Response:
This could certainly be a possibility, thank you. We have added this alternative hypothesis to the text (L144-145).

Comment:
L137: based on the M&M, it does not seem that different substitution models were systematically tested. What would be the best substitution model according to e.g., ModelFinder?

Response:
We thank the reviewer for this point. We did not perform a separate model-selection search on the reduced dataset used for the revised Fig 2 A analysis. Instead, we adopted the substitution-model (Q.pfam+R10+F model using 1,000 ultrafast bootstrap replicates, 1,000 SH-aLRT replicates) used in the broader Nucleocytoviricota phylogenomic benchmarking performed in Vasquez et al. (2025) that also included model test. We now cite this study explicitly in the Methods. We have also revised the text to avoid implying that multiple models were systematically compared within the present dataset.

Comment:
L228: could you please define in the text what Cali8-V and Cali8-NV refers to?

Response:
This section of the results has been dramatically revised, including clarifying the abbreviations used to describe fungal isolates (L277-278, 283-284). We also now identify in the Fig 3 legend that the "A" and "N" refer to "abnormal" and "normal" phenotypes.

Comment:
L697: DNA methylation detection is often trained on human or plant DNA methylation profiles. How suitable is Modkit in detecting mC (CpG) in fungal genomes?

Response:
In general, these modifications callings are robust and reliable. Please see the following papers for more about fungal 5mC detection:

- Schotanus K, Yadav V, Heitman J (2021) Epigenetic dynamics of centromeres and neocentromeres in *Cryptococcus deuterogattii*. PLOS Genetics 17(8): e1009743. https://doi.org/10.1371/journal.pgen.1009743
- Coelho MA, David-Palma M, Shea T, Bowers K, McGinley-Smith S, et al. (2024) Comparative genomics of the closely related fungal genera *Cryptococcus* and *Kwoniella* reveals karyotype dynamics and suggests evolutionary mechanisms of pathogenesis. PLOS Biology 22(6): e3002682. https://doi.org/10.1371/journal.pbio.3002682

And also the following regarding 6mA detection:

- Lax, C., Mondo, S.J., Osorio-Concepción, M. *et al.* Symmetric and asymmetric DNA N6-adenine methylation regulates different biological responses in Mucorales. *Nat Commun* 15, 6066 (2024). https://doi.org/10.1038/s41467-024-50365-2
- Romero Charria, P., Navarrete, C., Ovchinnikov, V. *et al.* Adenine DNA methylation associated with transcriptionally permissive chromatin is widespread across eukaryotes. *Nat Genet* 57, 3126–3136 (2025). https://doi.org/10.1038/s41588-025-02409-6

Comment:
Figure 3C: please add label to the plots to emphasis what is shown in the upper and lower plot. Are the other contigs in the lower panel with similar coverage to the one with the capsid protein also considered to be part of the virus? If this is the case, this should be highlighted.

Response:
Thank you. Figure 3 has been substantially revised, and this panel has been removed from the main text. This figure is now included in the supporting material as Fig S4. The upper and lower plots are now labeled to indicate the isolate of the genome assembly shown, and the figure legend describe that "The contigs in Cali8 with coverage of ~3.5k were identified as viral regions per the criteria outlined in the methods, and confirmed by chromosomal placement in the Nanopore assembly; the contigs with ~2k coverage are mitochondrial."

Comment:
Figure 4: many of the labels in the panels are not described in the figure legend. Please add these details.

Response:
Figure 4 has been removed in this revised version.

Reviewer 2:

Giant viruses (phylum Nucleocytoviricota) are a major group of large double-stranded DNA viruses that infect a wide range of eukaryotes and are abundant in various ecosystems. In this work, Myers et al. identified giant virus genome fragments in the nuclear genomes of zoosporic fungi and provide evidence for giant virus active infection and replication in fungal hosts. Overall, this study sheds light on the relationships between giant viruses and fungi and has important implications for their ecological interactions. My suggestions for the manuscript are as follows:

Comment:
1. The delineations of Mycodnaviridae and IM_02 can be more clearly described. In line 142, it seems that IM_02 is now dubbed Mycodnaviridae. Fig. 2 and its legend lines 162-163 also suggest this entire clade in Fig. 2A (though not labeled), including the three IM_02 GVMAGs, is Mycodnaviridae. However, the next sentence (lines 163-164) seems to say only those five viruses subtended by red branches are Mycodnaviridae. So this is quite confusing. Please indicate clearly in the main text, in the figure (label the whole Mycodnaviridae clade), and in the figure legend whether the newly defined Mycodnaviridae includes the three IM_02 GVMAGs and whether IM_02 is expanded to and now synonymous with the newly defined Mycodnaviridae. It would be tremendously helpful to have such clear definitions when the term Mycodnaviridae is used in the future.

Response:
Thank you for your close attention to these details. *IM_02* is indeed now dubbed *Mycodnaviridae*. This phylogenetic resolution is more thoroughly addressed in a forthcoming manuscript, including authors James and Schulz, the preprint of which can be found here: https://www.biorxiv.org/content/10.1101/2025.09.26.678796v3. We now reference this preprint in the text (L165).

We have updated Figure 2 with a label for *Mycodnaviridae*. We also clarified in the legend that the GVMAGs (previously *IM_02)* and the newly identified giant virus assemblies from fungi are together *Mycodnaviridae* (L163-165).

Comment:
2. Fig2A: the more basal branching topology is not easily legible. Please enlarge the figure and make the lines thin enough so they don't overlap. Line 164 mentions that the tree is rooted at Pokkesviricetes, but how come Pimascovirales (Megaviricetes) ends up on the same side with Pokkesviricetes (Chitovirales, Asfuvirales)?

Response:

We reran the phylogenetic analyses using the latest GVMAGs dataset, which improved the resolution of the tree and resulted in the correct topology as it is currently understood. Fig 2 A has been edited for legibility.

Comment:

3. Have the authors tried to test if spent media from fungal strains with productive infections or apparent VLPs cause signs of infection (viral DNA production, observation of VLPs, etc.) in other fungi that didn't have viruses?

Response:

Thank you for the suggestion! In response to this recommendation, we tested whether mating and growing uninfected *Allomyces* strains in the spent media of strains possessing the capacity for productive infection would result in virus transfer. Our qPCR results indicated that transfer did not occur, though there could be a number of reasons for this. Most critically, we have not yet found a reliable method for triggering virus activation. We describe this in the results (L298-300) and methods (L804-818).

Comment:

4. Abstract lines 32-34: "These viruses may have shaped the early evolution of these fungal lineages ..." ; line 318-319 suggests there is "a possible ancient association between fungi and giant viruses". What is the evidence that supports the idea that the relationships between giant viruses and fungi started early in time or have been ancient? It is not so clear how the notion of time weighed in here. It would be nice to include more comprehensive discussion on the how ancient giant viruses, fungi, and their relationships can be. Lines 315-317 mentions that fungi can be hosts to multiple distinct lineages of giant viruses. It can be interesting to note that animals are also hosts to at least three distinct giant virus clades, which can be the result of repeated adaptions by different giant virus lineages to animal hosts and not necessarily mean that early animal evolution was shaped by giant viruses.

Response:

Ancient associations seem very likely given the abundance of GVOGs found across the multiple phylum-level lineages of zoosporic fungi, the fact that Mycodnaviridae form a distinct clade (Supp. Table 1), and the hypothesis of GV origins near the time of eukaryogenesis. However, the reviewer raises a good point that the idea “viruses may have shaped the early evolution of these fungal lineages” is hardly the only possibility. Since the focus of this paper is to share the finding of a new clade of giant viruses in zoosporic fungi, rather than to characterize the historical relationship between giant viruses and the fungal kingdom, we have removed much of the “ancient association” language throughout this manuscript. However we keep in the discussion a paragraph about viruses motivating evolutionary innovation in which we cite a fungal cell cycle gene hypothesized to have evolved from viral transfer . We agree this is a fascinating topic, and the observation that animals, too, are host to numerous distinct clades inspires ideas for future exploration and thinking.

Reviewer 3

The manuscript "Discovery of giant viruses as past and present infections of zoosporic fungi" describes the discovery of full giant virus genome sequences from the Nucleocytoviricota phylum in fungi. This is an important finding describing discovery of the first known giant virus in fungi. Furthermore, and as the authors point out, only a very small number of DNA viruses have been discovered in fungi and this may relate to a bias towards the study of dikaryotic species. As this new discovery is in a non-dikaryotic species, it suggests continued investigation in this subkingdom may yield further advancements in the burgeoning mycovirology field. Using HMM searches of genome sequencing data, the authors identify MCPs diagnostic of Nucleocytoviricota. Leveraging this discovery, they expand the search for fungal Nucleocytoviricota MCPs to document their widespread occurrence in mostly non-dikaryotic species. Following phylogenetic analysis, they compare genomes from 5 apparently complete viruses of what they have termed "mycodnaviridae" to identify the core genes of this new family of giant viruses. Next the authors interrogate one virus from Allomyces they named A. arbusculus Burma1F that is apparently integrated into a chromosome. An Allomyces isolate named Cali8 is described and the authors present evidence that A. arbusculus Burma1F may be actively replicating in Cali8. EM images are shown to uphold the possibility that viral particles of A. arbusculus Burma1F are detected. While this is an exciting discovery, some of the claims are not fully upheld by the data. Moreover, the manuscript is at times nearly impossible to understand. Claims are made of potential phenotypic consequences of A. arbusculus Burma1F presence, but these are weak and it is a challenge to even understand what the authors are claiming about these findings. Despite the cautious language the authors use, these shortcomings are nevertheless an obstacle to publication in PLOS Biology. Further comments below.

Comment:

The Introduction section is terse and lacks a sorely needed description of giant viruses and what is known about them. Detrimental consequences of the absence of any such introduction to giant viruses reverberate throughout the manuscript. Is it known that giant virus genomes are riddled with genes from disparate organisms? What is known of the core set of Nucleocytoviricota genes? Are they typically known to integrate into the genomes and remain active? These and other basic matters of Nucleocytoviricota biology need to be presented in the introduction to make the manuscript accessible.

> **Response:**
> We have substantially altered the introduction and now include paragraphs describing giant viruses and their biology, which we agree was sorely needed (L54-89).We thank the reviewer for steering us toward this major improvement.

Comment:

-Figure 1. What exactly is 1A supposed to be showing? What distinguishes a "MCP contig" from mycodnaviridae in these panels?

Response:

Different flavors of endogenization were found, better showcased in Fig S2 (see lines 107-118). In a few isolates, singular MCP genes were found on otherwise entirely fungal-looking contigs. In other isolates, there appeared to be complete giant virus genomes within a fungal contig. To improve clarity, we have updated figure 1 and removed the “MCP contig” boxes. Now, we much more clearly show the differences in genome characteristics between fungal contigs and those which we identified as *Mycodnaviridae*.

Comment:

Line 101. Why are there 2 contigs for A. arbusculus Burma1F? This is not elaborated on and is confusing.

Response:

This figure is based on the Illumina data from which we originally identified the virus. We later generated a long-read assembly confirming that these two contigs from the original assembly are side-by-side. We articulated this for clarity in L202-203.

Comment:

The evidence for active viral replication is unconvincing. The authors show integrated A. arbusculus *Burma1F and do an admirable job describing the chromosomal landscape of this organism, while providing evidence that* A. arbusculus *Burma1F is dormant, with no corresponding RNA detected. They rely on increased DNA read counts discovered in Cali8 as evidence of viral replication. This may be true but much more should be done to uphold this conclusion. RNA reads should now be detectable for example. Moreover, viral particles should be purified to confirm* A. arbusculus *Burma1F replication and particle production. These particles can be interrogated for the presence of the A. arbusculus Burma1F genome. The EM approach is too speculative and FISH may be a better approach to identify replicating* A. arbusculus *Burma1F in situ.*

Response:

Thank you for these very thoughtful comments and suggestions. We conducted some additional experiments to test for viral replication, including additional RNA-seq, qPCR of tissues at various life cycle stages and environmental conditions, and qPCR of cDNA to assess viral gene expression. These results are now described (L254-300) and add additional lines of evidence in support of active viral replication, which is inducible, though somewhat unpredictably, by high temperature.

We agree that at this stage our EM work is premature. As described above, we have decided to remove this result until we are able to isolate and sequence particles. We also agree that FISH would be ideal. These two directions will require investment beyond the scope of this paper, but we look forward to pursuing them.

Comment:
The data presented in 3C and 3D are not clearly explained. The section of the manuscript describing the data shown in 3E and 3F are impossible to discern. I read this over 4 times and still have no idea what was done and what the claims are. What does it mean that Cali8 shows "sectoring" at 42˚? How does this relate to isolating Cali8-N and Cali8-V? The rest of this paragraph is extremely difficult to follow. I have no way to evaluate the claims, and I'm not even sure exactly what the claims are.

Response:
We have removed these two panels from Figure 3 and heavily revised this section of the results for both clarity and content (L254-308).
