# Supplementary material for: Mycodnaviridae is a clade of giant viruses that persistently infect zoosporic fungi
Source: PLoS Biol. 2026 Aug 25;24(8):e3003937. doi: 10.1371/journal.pbio.3003937 (PMC13533422; doi:10.1371/journal.pbio.3003937)
Supplement: Supplementary file 10 [file pbio.3003937.s010.pdf]

"Mycodnaviridae are a clade of giant viruses that persistently infect zoosporic fungi"

Jillian M. Myers, Frederik Schulz, Saleh Rahimlou, Vikas Yadav, Kevin R. Amses, D. Rabern Simmons, Sheng Sun, Michelle Orozco-Quime, Joseph Heitman, Jason E. Stajich, Timothy Y. James

Dear Reviewers,

We are again grateful for your time reading our manuscript on giant viruses in fungi, for your close attention to detail and continued thoughtful advice. Below are details of how we have addressed each comment.

Please note that in addition to your comments, we updated Fig1A to include individual data points in addition to the boxplots in alignment with data presentation best practices. We have also added all experimental data to the University of Michigan Deep Blue repository. Finally, we updated the title of the manuscript per recommendations from the journal to better suit the broad readership.

Reviewer 1:

The resubmission of the manuscript 'Discovery of Mycodnaviridae, a clade of giant viruses that persistently infect zoosporic fungi' by Myers and colleagues reports on genomics, phylogenetics, and microscopic research to detect and characterize giant viruses in zoosporic fungi. While the resubmission successfully addresses my main concerns raised by the initial manuscript that had focused on technical aspects, several comments related to the clarity of the current submission remain. I would encourage the authors to address these comments and to further consider the diverse readership of PLoS Biology when revising their submission to further enhance accessibility and clarity throughout the submission.

Few examples that necessitate the authors attention:

L65: 'Despite their incredible abundance, relatively few have been linked to a host…' please specify what is referred to here. I assume giant viruses but that could be made clearer.

Response:

Thank you for this catch. This now reads "...relatively few GVs have been linked to a host…"

L84: what does EVE stand for? Please clarify.

Response:

Thanks again for your close attention! We now define this abbreviation in this first appearance in the text.

L91: it would be nice if the introduction would close with a 1-line outline what the manuscript here will address and how it relates to the knowledge gap.

Response:

We have appended some context to prime the reader for what is to come. These lines (L89-91) now read "Active GV infections in fungi have not previously been documented nor have novel clades that include active giant viruses in fungi been characterized; we describe both here and present a promising new model system to further examine GV-fungal interactions."

L96: please provide details what fungi where queried here

Response:

In this text we point the reader to Fig S1 and Table S1 where we provide detailed information (phylogenetic, taxonomic, and isolate/strain) about the fungi queried. We feel this should be sufficient.

L106: I assume the number (58) refers to the Allomyces isolate

Response:

Thank you for identifying a point of unclarity. "30/58" is meant to articulate "thirty out of fifty-eight unique isolates" that were found to be positive for the viral capsid by PCR. We have clarified this in the text.

L282: '…original strain ("Parental strain") and strains with abnormal ("Sub 1-A") and 282 normal phenotype ("Sub 2-N")…' it seems that the relevance of the different strains is not clearly explained here (only much later in L322ff). Moreover, the coverage plots are not labeled accordingly, and it is thus challenging to match the description to the correct panel elements. Please clarify

Response:

We added the word "subculture" before describing the strains in this figure legend to help clarify the naming of "Sub #A/N" phenotypes. Both the coverage plots and RNA-seq read plots are labeled according to the strain. Note that these only apply to panel D. In panel B, the coverage plot is not labeled because this was a different culture altogether (*A. arbusculus* B1F, rather than *A. arbusculus* Cali8 in panel D), for which only one strain was sequenced.

L305: please clarify to which S-Figure this text refers to

Response:

Thank you again for your close attention! We have fixed this error.

<u>Reviewer 2:</u>

The authors have fully addressed the previous comments.
Just one typo in Fig. 2: "Mycovdnaviridae" should be Mycodnaviridae.

Response:

Thank you for catching this! It would have haunted me if I'd found this in the published version.

Reviewer 3:

The authors have satisfactorily addressed my comments in this revision.

Response:
We thank you again for your role in the improvement of this manuscript.
