# Supplementary material for: Mycodnaviridae is a clade of giant viruses that persistently infect zoosporic fungi
Source: PLoS Biol. 2026 Aug 25;24(8):e3003937. doi: 10.1371/journal.pbio.3003937 (PMC13533422; doi:10.1371/journal.pbio.3003937)
Supplement: Peer review history

# Editor report: Decision Letter 0

**DOI:** 10.1371/journal.pbio.3003937.r001

**Authors:** Melissa Vazquez Hernandez

19 Jun 2025

Dear Jill,

Thank you for submitting your manuscript entitled "Discovery of giant viruses as past and present infections of zoosporic fungi" for consideration by PLOS Biology.

Your manuscript has now been evaluated by the PLOS Biology editorial staff, as well as by an academic editor with relevant expertise, and I am writing to let you know that we would like to send your submission out for external peer review as a Discovery Report.

However, before we can send your manuscript to reviewers, we need you to complete your submission by providing the metadata that is required for full assessment. To this end, please login to Editorial Manager where you will find the paper in the 'Submissions Needing Revisions' folder on your homepage. Please click 'Revise Submission' from the Action Links and complete all additional questions in the submission questionnaire. Please, when adding the rest of the metadata choose "Short Report".

Once your full submission is complete, your paper will undergo a series of checks in preparation for peer review. After your manuscript has passed the checks it will be sent out for review. To provide the metadata for your submission, please Login to Editorial Manager (https://www.editorialmanager.com/pbiology) within two working days, i.e. by Jun 21 2025 11:59PM.

If your manuscript has been previously peer-reviewed at another journal, PLOS Biology is willing to work with those reviews in order to avoid re-starting the process. Submission of the previous reviews is entirely optional and our ability to use them effectively will depend on the willingness of the previous journal to confirm the content of the reports and share the reviewer identities. Please note that we reserve the right to invite additional reviewers if we consider that additional/independent reviewers are needed, although we aim to avoid this as far as possible. In our experience, working with previous reviews does save time.

If you would like us to consider previous reviewer reports, please edit your cover letter to let us know and include the name of the journal where the work was previously considered and the manuscript ID it was given. In addition, please upload a response to the reviews as a 'Prior Peer Review' file type, which should include the reports in full and a point-by-point reply detailing how you have or plan to address the reviewers' concerns.

During the process of completing your manuscript submission, you will be invited to opt-in to posting your pre-review manuscript as a bioRxiv preprint. Visit http://journals.plos.org/plosbiology/s/preprints for full details. If you consent to posting your current manuscript as a preprint, please upload a single Preprint PDF.

Feel free to email us at plosbiology@plos.org if you have any queries relating to your submission.

Best wishes,

Melissa

Melissa Vazquez Hernandez, Ph.D.

Associate Editor

PLOS Biology

mvazquezhernandez@plos.org

# Peer review: Decision Letter 1

**DOI:** 10.1371/journal.pbio.3003937.r002

**Authors:** Melissa Vazquez Hernandez

1 Aug 2025

Dear Dr Myers,

Thank you for your patience while your manuscript "Discovery of giant viruses as past and present infections of zoosporic fungi" was peer-reviewed at PLOS Biology. Your manuscript has been evaluated by the PLOS Biology editors, an Academic Editor with relevant expertise, and by three independent reviewers.

As you will see in the reviewer reports, although the reviewers acknowledge the potential interest in your findings, they have also raised a substantial number of crucial concerns. Based on their specific comments and following discussion with the Academic Editor, it is clear that a substantial amount of work would be required to meet the criteria for publication in PLOS Biology. Given our and the reviewer interest in your study, we would be open to inviting a comprehensive revision of the work; however, this would need to thoroughly address all the reviewers' comments. Reviewer 1 has some concerns regarding the bioinformatic analyses and requests that some of these are repeated. Reviewer 2 highlights some points that will require modification to the text and the figures. The reviewer also suggests to evaluate if the media may be causing signs of infections. Reviewer 3 thinks this is an exciting discovery but that some claims are not supported, for example the evidence for active viral replication. A successful revision will need to strengthen the claims following the reviewers' suggestions, improve the manuscript's structure for enhanced readability, and address other relevant concerns regarding wording and presentation.

In addition to these revisions, you will need to complete some formatting changes, which you will receive in a follow up email. A member of our team will be in touch with a set of requests shortly.

We appreciate that these requests represent a great deal of extra work, and we are willing to relax our standard revision time to allow you 6 months to revise your study. Please email us (plosbiology@plos.org) if you have any questions or concerns, or envision needing a (short) extension.

At this stage, your manuscript remains formally under active consideration at our journal; please notify us by email if you do not intend to submit a revision so that we may withdraw it.

**IMPORTANT - SUBMITTING YOUR REVISION**

Your revisions should address the specific points made by each reviewer. Please submit the following files along with your revised manuscript:

1. A 'Response to Reviewers' file - this should detail your responses to the editorial requests, present a point-by-point response to all of the reviewers' comments, and indicate the changes made to the manuscript.

*NOTE: In your point by point response to the reviewers, please provide the full context of each review. Do not selectively quote paragraphs or sentences to reply to. The entire set of reviewer comments should be present in full and each specific point should be responded to individually, point by point.

You should also cite any additional relevant literature that has been published since the original submission and mention any additional citations in your response.

2. In addition to a clean copy of the manuscript, please also upload a 'track-changes' version of your manuscript that specifies the edits made. This should be uploaded as a "Revised Article with Changes Highlighted " file type.

*Resubmission Checklist*

When you are ready to resubmit your revised manuscript, please refer to this resubmission checklist: https://plos.io/Biology_Checklist

To submit a revised version of your manuscript, please go to https://www.editorialmanager.com/pbiology/ and log in as an Author. Click the link labelled 'Submissions Needing Revision' where you will find your submission record.

Please make sure to read the following important policies and guidelines while preparing your revision:

*Published Peer Review*

Please note while forming your response, if your article is accepted, you may have the opportunity to make the peer review history publicly available. The record will include editor decision letters (with reviews) and your responses to reviewer comments. If eligible, we will contact you to opt in or out. Please see here for more details:

https://blogs.plos.org/plos/2019/05/plos-journals-now-open-for-published-peer-review/

*PLOS Data Policy*

Please note that as a condition of publication PLOS' data policy (http://journals.plos.org/plosbiology/s/data-availability) requires that you make available all data used to draw the conclusions arrived at in your manuscript. If you have not already done so, you must include any data used in your manuscript either in appropriate repositories, within the body of the manuscript, or as supporting information (N.B. this includes any numerical values that were used to generate graphs, histograms etc.). For an example see here: http://www.plosbiology.org/article/info%3Adoi%2F10.1371%2Fjournal.pbio.1001908#s5

*Blot and Gel Data Policy*

We require the original, uncropped and minimally adjusted images supporting all blot and gel results reported in an article's figures or Supporting Information files. We will require these files before a manuscript can be accepted so please prepare them now, if you have not already uploaded them. Please carefully read our guidelines for how to prepare and upload this data: https://journals.plos.org/plosbiology/s/figures#loc-blot-and-gel-reporting-requirements

*Protocols deposition*

To enhance the reproducibility of your results, we recommend that if applicable you deposit your laboratory protocols in protocols.io, where a protocol can be assigned its own identifier (DOI) such that it can be cited independently in the future. Additionally, PLOS ONE offers an option for publishing peer-reviewed Lab Protocol articles, which describe protocols hosted on protocols.io. Read more information on sharing protocols at https://plos.org/protocols?utm_medium=editorial-email&utm_source=authorletters&utm_campaign=protocols

Thank you again for your submission to our journal. We hope that our editorial process has been constructive thus far, and we welcome your feedback at any time. Please don't hesitate to contact us if you have any questions or comments.

Sincerely,

Melissa

Melissa Vazquez Hernandez, Ph.D.

Associate Editor

PLOS Biology

mvazquezhernandez@plos.org

-----------------------------------------

REVIEWERS' COMMENTS:

-----------------------------------------

Reviewer #1:

Viruses are among the terra incognita in biology. Research into viruses in many eukaryotic lineages has only recently been accelerated by rapid advances in (meta)genomics. In the fungal kingdom, research into viruses has been lacking behind other eukaryotic lineages, and DNA and RNA viruses have only recently been detected, underscoring the tremendous knowledge gaps surrounding viruses in fungi.

The short report 'Discovery of giant viruses as past and present infections of zoosporic fungi' by Myers and colleagues report on genomics, phylogenetics, and microscopic research to detect and characterize giant viruses in zoosporic fungi. The manuscript aims to address a timely and interesting research question, and the general approach (experimental design) is reasonable; all data needed to replicate the work is present in the public domain or has been deposited by the authors. However, I have several concerns and suggestions on the performed analyses, the robustness of the results, and the clarity of the manuscript that need to be addresses.

The bioinformatics analyses need clarification and reanalyses:

-it is not clear why the manuscript first reports MCP in a small subset of fungi and only later queries a bigger dataset (Mycocosm; L69ff). If seems more logical and in line with the aim of the article if the authors would have queried the more comprehensive dataset from the start.

-MCP homologs are initially identified using HMMer searches but Mycocosm as well as the metagenomic data is queried via (bidirectional) blast. To detect remote homology and capture the breadth of these viruses throughout fungi, the latter searches need to be repeated via more sensitive HMMer searches and/or via profile-vs-profile searches.

-it is not clear from the manuscript if all genomes and their contigs are scanned for signals of viral contigs with ViralRecall. It is also unclear how the size of the viruses is determined; is it the entire size of a contig? This could be problematic given the chimeric origin of the genes reported on these contig, suggesting that these viral sequences rapidly change. Lastly, algorithms to detect viral contigs differ significantly (see e.g., Wu et al. 2024). How would the application of different algorithms to classify viral contigs impact the manuscript's results?

-in L105, tetramer frequency is used to bin contigs. Are the binning results also supported by read coverage, e.g., by mapping the original sequencing data back to viral bins?

It is mentioned in the manuscript that MCP in fungal genomes are indications of ancient viral association / infections, which then would also explain the mixed origins of genes associated with the viruses as well as differences in GC content etc. It is shown, but not further discussed, that MCP and viral contigs have less introns that the average fungus. Is it known if fungal viruses have introns? The presence of some introns in viral sequences might provide additional evidence to when viruses entered the hosts. Figure 3B shows the read coverage of contig3 with viral sequence embedded. The coverage at the virus seems to be less than the remainder of the contig. Could the authors please provide an explanation for this observation?

While a short report is surely a suitable venue for these observations, the rationale for some experiments is currently very challenging to follow and details to contextualize the results are missing. To enhance clarity, the results and discussion section should be merged, additional background needs to be added (see also comments below), the motivation for individual experiments (e.g., L77) need to be better explained, and solid results need to be clearer separated from hypothesis or assumptions, e.g., in the abstract, L237, or L183; the presented data shows that the virus is not expressed but does not provide evidence that the host actively silences the virus.

Additional comments/suggestions:

L57: it is not clear what fungal homologs have been identified in viral metagenomes. Please clarify this statement.

L60: please provide more details how the evidence differs, and what the authors refer to as 'robustness'

L91: the implication of this observation remains unclear and needs to be better explained.

L112: what is the authors' evidence that the two viruses are derived from the parents. Could these not be merely the result of a co-infection?

L137: based on the M&M, it does not seem that different substitution models were systematically tested. What would be the best substitution model according to e.g., ModelFinder?

L228: could you please define in the text what Cali8-V and Cali8-NV refers to?

L697: DNA methylation detection is often trained on human or plant DNA methylation profiles. How suitable is Modkit in detecting mC (CpG) in fungal genomes?

Figure 3C: please add label to the plots to emphasis what is shown in the upper and lower plot. Are the other contigs in the lower panel with similar coverage to the one with the capsid protein also considered to be part of the virus? If this is the case, this should be highlighted.

Figure 4: many of the labels in the panels are not described in the figure legend. Please add these details.

-----------------------------------------

Reviewer #2:

Giant viruses (phylum Nucleocytoviricota) are a major group of large double-stranded DNA viruses that infect a wide range of eukaryotes and are abundant in various ecosystems. In this work, Myers et al. identified giant virus genome fragments in the nuclear genomes of zoosporic fungi and provide evidence for giant virus active infection and replication in fungal hosts. Overall, this study sheds light on the relationships between giant viruses and fungi and has important implications for their ecological interactions. My suggestions for the manuscript are as follows:

1. The delineations of Mycodnaviridae and IM_02 can be more clearly described. In line 142, it seems that IM_02 is now dubbed Mycodnaviridae. Fig. 2 and its legend lines 162-163 also suggest this entire clade in Fig. 2A (though not labeled), including the three IM_02 GVMAGs, is Mycodnaviridae. However, the next sentence (lines 163-164) seems to say only those five viruses subtended by red branches are Mycodnaviridae. So this is quite confusing. Please indicate clearly in the main text, in the figure (label the whole Mycodnaviridae clade), and in the figure legend whether the newly defined Mycodnaviridae includes the three IM_02 GVMAGs and whether IM_02 is expanded to and now synonymous with the newly defined Mycodnaviridae. It would be tremendously helpful to have such clear definitions when the term Mycodnaviridae is used in the future.

2. Fig2A: the more basal branching topology is not easily legible. Please enlarge the figure and make the lines thin enough so they don't overlap. Line 164 mentions that the tree is rooted at Pokkesviricetes, but how come Pimascovirales (Megaviricetes) ends up on the same side with Pokkesviricetes (Chitovirales, Asfuvirales)?

3. Have the authors tried to test if spent media from fungal strains with productive infections or apparent VLPs cause signs of infection (viral DNA production, observation of VLPs, etc.) in other fungi that didn't have viruses?

4. Abstract lines 32-34: "These viruses may have shaped the early evolution of these fungal lineages ..." ; line 318-319 suggests there is "a possible ancient association between fungi and giant viruses". What is the evidence that supports the idea that the relationships between giant viruses and fungi started early in time or have been ancient? It is not so clear how the notion of time weighed in here. It would be nice to include more comprehensive discussion on the how ancient giant viruses, fungi, and their relationships can be. Lines 315-317 mentions that fungi can be hosts to multiple distinct lineages of giant viruses. It can be interesting to note that animals are also hosts to at least three distinct giant virus clades, which can be the result of repeated adaptions by different giant virus lineages to animal hosts and not necessarily mean that early animal evolution was shaped by giant viruses.

-----------------------------------------

Reviewer #3:

The manuscript "Discovery of giant viruses as past and present infections of zoosporic fungi" describes the discovery of full giant virus genome sequences from the Nucleocytoviricota phylum in fungi. This is an important finding describing discovery of the first known giant virus in fungi. Furthermore, and as the authors point out, only a very small number of DNA viruses have been discovered in fungi and this may relate to a bias towards the study of dikaryotic species. As this new discovery is in a non-dikaryotic species, it suggests continued investigation in this subkingdom may yield further advancements in the burgeoning mycovirology field. Using HMM searches of genome sequencing data, the authors identify MCPs diagnostic of Nucleocytoviricota. Leveraging this discovery, they expand the search for fungal Nucleocytoviricota MCPs to document their widespread occurrence in mostly non-dikaryotic species. Following phylogenetic analysis, they compare genomes from 5 apparently complete viruses of what they have termed "mycodnaviridae" to identify the core genes of this new family of giant viruses. Next the authors interrogate one virus from Allomyces they named A. arbusculus Burma1F that is apparently integrated into a chromosome. An Allomyces isolate named Cali8 is described and the authors present evidence that A. arbusculus Burma1F may be actively replicating in Cali8. EM images are shown to uphold the possibility that viral particles of A. arbusculus Burma1F are detected. While this is an exciting discovery, some of the claims are not fully upheld by the data. Moreover, the manuscript is at times nearly impossible to understand. Claims are made of potential phenotypic consequences of A. arbusculus Burma1F presence, but these are weak and it is a challenge to even understand what the authors are claiming about these findings. Despite the cautious language the authors use, these shortcomings are nevertheless an obstacle to publication in PLOS Biology. Further comments below.

The Introduction section is terse and lacks a sorely needed description of giant viruses and what is known about them. Detrimental consequences of the absence of any such introduction to giant viruses reverberate throughout the manuscript. Is it known that giant virus genomes are riddled with genes from disparate organisms? What is known of the core set of Nucleocytoviricota genes? Are they typically known to integrate into the genomes and remain active? These and other basic matters of Nucleocytoviricota biology need to be presented in the introduction to make the manuscript accessible.

-Figure 1. What exactly is 1A supposed to be showing? What distinguishes a "MCP contig" from mycodnaviridae in these panels?

Line 101. Why are there 2 contigs for A. arbusculus Burma1F? This is not elaborated on and is confusing.

The evidence for active viral replication is unconvincing. The authors show integrated A. arbusculus Burma1F and do an admirable job describing the chromosomal landscape of this organism, while providing evidence that A. arbusculus Burma1F is dormant, with no corresponding RNA detected. They rely on increased DNA read counts discovered in Cali8 as evidence of viral replication. This may be true but much more should be done to uphold this conclusion. RNA reads should now be detectable for example. Moreover, viral particles should be purified to confirm A. arbusculus Burma1F replication and particle production. These particles can be interrogated for the presence of the A. arbusculus Burma1F genome. The EM approach is too speculative and FISH may be a better approach to identify replicating A. arbusculus Burma1F in situ.

The data presented in 3C and 3D are not clearly explained. The section of the manuscript describing the data shown in 3E and 3F are impossible to discern. I read this over 4 times and still have no idea what was done and what the claims are. What does it mean that Cali8 shows "sectoring" at 42˚? How does this relate to isolating Cali8-N and Cali8-V? The rest of this paragraph is extremely difficult to follow. I have no way to evaluate the claims, and I'm not even sure exactly what the claims are.

# Author comment: Author response to Decision Letter 2

**DOI:** 10.1371/journal.pbio.3003937.r003

6 May 2026

Attachment Submitted filename: Mycodnaviridae-Response-to-Reviewers.pdf

# Peer review: Decision Letter 2

**DOI:** 10.1371/journal.pbio.3003937.r004

**Authors:** Melissa Vazquez Hernandez

17 Jun 2026

Dear Dr Myers,

Thank you for your patience while we considered your revised manuscript "Discovery of Mycodnaviridae, a clade of giant viruses that persistently infect zoosporic fungi" for consideration as a Short Reports at PLOS Biology. Your revised study has now been evaluated by the PLOS Biology editors, the Academic Editor and the original reviewers.

In light of the reviews, which you will find at the end of this email, we are pleased to offer you the opportunity to address one last concern in a revision that we anticipate should not take you very long. We will then assess your revised manuscript and your response to the reviewers' comments with our Academic Editor aiming to avoid further rounds of peer-review.

As you will see in the reports, while the reviewers are now satisfied with the revision, one concern remains regarding the accesibility of the text. After discussing with the Academic Editor, given the broad readership of PLOS Biology, we require some modifications on the text to make it more accesible. Reviewer 1 has given excellent suggestions to start with. Please also address the following editorial points:

1) We routinely suggest changes to titles to ensure maximum accessibility for a broad, non-specialist readership, and to ensure they reflect the contents of the paper. In this case, we would suggest some changes, as follows. Please ensure you change both the manuscript file and the online submission system, as they need to match for final acceptance:

"Mycodnaviridae are a clade of giant viruses that persistently infect zoosporic fungi"

2) Please add the weblink of the funding agencies in the Financial Disclosure statement in the manuscript details during submission as well as in your text.

3) Please add to your Competing Interests the following statement “JH is a member of PLOS Biology’s Editorial Board. The other authors declare that no competing interests exist."

4) You may be aware of the PLOS Data Policy, which requires that all data be made available without restriction: http://journals.plos.org/plosbiology/s/data-availability. For more information, please also see this editorial: http://dx.doi.org/10.1371/journal.pbio.1001797

Please supply the numerical values either in the a supplementary file or as a permanent DOI’d deposition for the following figures:

Figure 1AC, 4FG, S4

NOTE: the numerical data provided should include all replicates AND the way in which the plotted mean and errors were derived (it should not present only the mean/average values).

5) Please cite the location of the data clearly in all relevant main and supplementary Figure legends, e.g. “The data underlying this Figure can be found in S1 Data” or “The data underlying this Figure can be found in https://doi.org/10.5281/zenodo.XXXXX”

6) Please provide the tree files for the phylogenetic trees in Figures 2A, S1, S6. Please make sure all relevant figures have scale bars.

7) Please add a scale bar in the all microscopy pictures

8) Supplementary files (e.g., excel). Please ensure that all data files are uploaded as 'Supporting Information' and are invariably referred to (in the manuscript, figure legends, and the Description field when uploading your files) using the following format verbatim: S1 Data, S2 Data, etc. Multiple panels of a single or even several figures can be included as multiple sheets in one excel file that is saved using exactly the following convention: S1_Data.xlsx (using an underscore).

9) Please ensure that your Data Statement in the submission system accurately describes where your data can be found and is in final format, as it will be published as written there

10) Per journal policy, if you have generated any custom code during the course of this investigation, please make it available without restrictions. Please ensure that the code is sufficiently well documented and reusable, and that your Data Statement in the Editorial Manager submission system accurately describes where your code can be found. More information on our Code Policy, what and how to share can be found here: https://journals.plos.org/plosbiology/s/code-availability

Please note that we cannot accept sole deposition of code in GitHub, as this could be changed after publication. However, you can archive this version of your publicly available GitHub code to Zenodo. Once you do this, it will generate a DOI number, which you will need to provide in the Data Accessibility Statement (you are welcome to also provide the GitHub access information). See the process for doing this here: https://docs.github.com/en/repositories/archiving-a-github-repository/referencing-and-citing-content

In addition to these revisions, you may need to complete some formatting changes, which you will receive in a follow up email. A member of our team will be in touch with a set of requests shortly. If you do not receive a separate email within a few days, please assume that checks have been completed, and no additional changes are required.

We expect to receive your revised manuscript within 1 month. Please email us (plosbiology@plos.org) if you have any questions or concerns, or would like to request an extension.

At this stage, your manuscript remains formally under active consideration at our journal; please notify us by email if you do not intend to submit a revision so that we withdraw the manuscript.

**IMPORTANT - SUBMITTING YOUR REVISION**

Your revisions should address the specific points made by each reviewer. Please submit the following files along with your revised manuscript:

1. A 'Response to Reviewers' file - this should detail your responses to the editorial requests, present a point-by-point response to all of the reviewers' comments, and indicate the changes made to the manuscript.

*NOTE: In your point-by-point response to the reviewers, please provide the full context of each review. Do not selectively quote paragraphs or sentences to reply to. The entire set of reviewer comments should be present in full and each specific point should be responded to individually.

You should also cite any additional relevant literature that has been published since the original submission and mention any additional citations in your response.

2. In addition to a clean copy of the manuscript, please also upload a 'track-changes' version of your manuscript that specifies the edits made. This should be uploaded as a "Revised Article with Changes Highlighted " file type.

*Resubmission Checklist*

When you are ready to resubmit your revised manuscript, please refer to this resubmission checklist: https://plos.io/Biology_Checklist

To submit a revised version of your manuscript, please go to https://www.editorialmanager.com/pbiology/ and log in as an Author. Click the link labelled 'Submissions Needing Revision' where you will find your submission record.

Please make sure to read the following important policies and guidelines while preparing your revision:

*Published Peer Review*

Please note while forming your response, if your article is accepted, you may have the opportunity to make the peer review history publicly available. The record will include editor decision letters (with reviews) and your responses to reviewer comments. If eligible, we will contact you to opt in or out. Please see here for more details:

https://blogs.plos.org/plos/2019/05/plos-journals-now-open-for-published-peer-review/

*PLOS Data Policy*

Please note that as a condition of publication PLOS' data policy (http://journals.plos.org/plosbiology/s/data-availability) requires that you make available all data used to draw the conclusions arrived at in your manuscript. If you have not already done so, you must include any data used in your manuscript either in appropriate repositories, within the body of the manuscript, or as supporting information (N.B. this includes any numerical values that were used to generate graphs, histograms etc.). For an example see here: http://www.plosbiology.org/article/info%3Adoi%2F10.1371%2Fjournal.pbio.1001908#s5

*Blot and Gel Data Policy*

We require the original, uncropped and minimally adjusted images supporting all blot and gel results reported in an article's figures or Supporting Information files. We will require these files before a manuscript can be accepted so please prepare them now, if you have not already uploaded them. Please carefully read our guidelines for how to prepare and upload this data: https://journals.plos.org/plosbiology/s/figures#loc-blot-and-gel-reporting-requirements

*Protocols deposition*

To enhance the reproducibility of your results, we recommend that if applicable you deposit your laboratory protocols in protocols.io, where a protocol can be assigned its own identifier (DOI) such that it can be cited independently in the future. Additionally, PLOS ONE offers an option for publishing peer-reviewed Lab Protocol articles, which describe protocols hosted on protocols.io. Read more information on sharing protocols at https://plos.org/protocols?utm_medium=editorial-email&utm_source=authorletters&utm_campaign=protocols

Thank you again for your submission to our journal. We hope that our editorial process has been constructive thus far, and we welcome your feedback at any time. Please don't hesitate to contact us if you have any questions or comments.

Sincerely,

Melissa

Melissa Vazquez Hernandez, Ph.D.

Associate Editor

PLOS Biology

mvazquezhernandez@plos.org

----------------------------------------------------------------

REVIEWERS' COMMENTS:

----------------------------------------------------------------

Reviewer #1:

The resubmission of the manuscript 'Discovery of Mycodnaviridae, a clade of giant viruses that persistently infect zoosporic fungi' by Myers and colleagues reports on genomics, phylogenetics, and microscopic research to detect and characterize giant viruses in zoosporic fungi. While the resubmission successfully addresses my main concerns raised by the initial manuscript that had focused on technical aspects, several comments related to the clarity of the current submission remain. I would encourage the authors to address these comments and to further consider the diverse readership of PLoS Biology when revising their submission to further enhance accessibility and clarity throughout the submission.

Few examples that necessitate the authors attention:

L65: 'Despite their incredible abundance, relatively few have been linked to a host…' please specify what is referred to here. I assume giant viruses but that could be made clearer.

L84: what does EVE stand for? Please clarify.

L91: it would be nice if the introduction would close with a 1-line outline what the manuscript here will address and how it relates to the knowledge gap.

L96: please provide details what fungi where queried here

L106: I assume the number (58) refers to the Allomyces isolate

L282: '…original strain ("Parental strain") and strains with abnormal ("Sub 1-A") and

282 normal phenotype ("Sub 2-N")…' it seems that the relevance of the different strains is not clearly explained here (only much later in L322ff). Moreover, the coverage plots are not labeled accordingly, and it is thus challenging to match the description to the correct panel elements. Please clarify

L305: please clarify to which S-Figure this text refers to

----------------------------------------------------------------

Reviewer #2:

The author's have fully addressed the previous comments.

Just one typo in Fig. 2: "Mycovdnaviridae" should be Mycodnaviridae.

----------------------------------------------------------------

Reviewer #3:

The authors have satisfactorily addressed my comments in this revision.

# Author comment: Author response to Decision Letter 3

**DOI:** 10.1371/journal.pbio.3003937.r005

8 Jul 2026

Attachment Submitted filename: Response to Reviewers_July26.pdf

# Editor report: Decision Letter 3

**DOI:** 10.1371/journal.pbio.3003937.r006

**Authors:** Melissa Vazquez Hernandez

23 Jul 2026

Dear Jill,

Thank you for the submission of your revised Short Reports "Mycodnaviridae are a clade of giant viruses that persistently infect zoosporic fungi" for publication in PLOS Biology. On behalf of my colleagues and the Academic Editor, Sarah Zanders, I am pleased to say that we can in principle accept your manuscript for publication, provided you address any remaining formatting and reporting issues. These will be detailed in an email you should receive within 2-3 business days from our colleagues in the journal operations team; no action is required from you until then. Please note that we will not be able to formally accept your manuscript and schedule it for publication until you have completed any requested changes.

Please take a minute to log into Editorial Manager at http://www.editorialmanager.com/pbiology/, click the "Update My Information" link at the top of the page, and update your user information to ensure an efficient production process.

PRESS

We frequently collaborate with press offices. If your institution or institutions have a press office, please notify them about your upcoming paper at this point, to enable them to help maximise its impact. If the press office is planning to promote your findings, we would be grateful if they could coordinate with biologypress@plos.org. If you have previously opted in to the early version process, we ask that you notify us immediately of any press plans so that we may opt out on your behalf.

We also ask that you take this opportunity to read our Embargo Policy regarding the discussion, promotion and media coverage of work that is yet to be published by PLOS. As your manuscript is not yet published, it is bound by the conditions of our Embargo Policy. Please be aware that this policy is in place both to ensure that any press coverage of your article is fully substantiated and to provide a direct link between such coverage and the published work. For full details of our Embargo Policy, please visit http://www.plos.org/about/media-inquiries/embargo-policy/.

Thank you again for choosing PLOS Biology for publication and supporting Open Access publishing. We look forward to publishing your study.

Sincerely,

Melissa

Melissa Vazquez Hernandez, Ph.D., Ph.D.

Senior Editor

PLOS Biology

mvazquezhernandez@plos.org
